# Influence of Crystallization Kinetics and Flow Behavior on Structural Inhomogeneities in 3D-Printed Parts Made from Semi-Crystalline Polymers

**DOI:** 10.1021/acs.macromol.3c01940

**Published:** 2024-03-19

**Authors:** Rene Sattler, Rui Zhang, Gaurav Gupta, Mengxue Du, Paul-Maximilian Runge, Holm Altenbach, René Androsch, Mario Beiner

**Affiliations:** † Fraunhofer Institute for Microstructure of Materials and Systems IMWS, Walter-Hülse-Str. 1, DE-06120 Halle (Saale), Germany; ‡ Faculty of Natural Sciences II, Martin-Luther-University Halle-Wittenberg, Heinrich-Damerow-Str. 4, D-06120 Halle (Saale), Germany; § Interdisciplinary Center for Transfer-Oriented Research in Natural Sciences, Martin-Luther-University Halle-Wittenberg, Universitätsplatz 10, D-06120 Halle (Saale), Germany; ∥ Institute of Mechanics, Otto-von-Guericke-University Magdeburg, Universitätsplatz 2, D-39106 Magdeburg, Germany

## Abstract

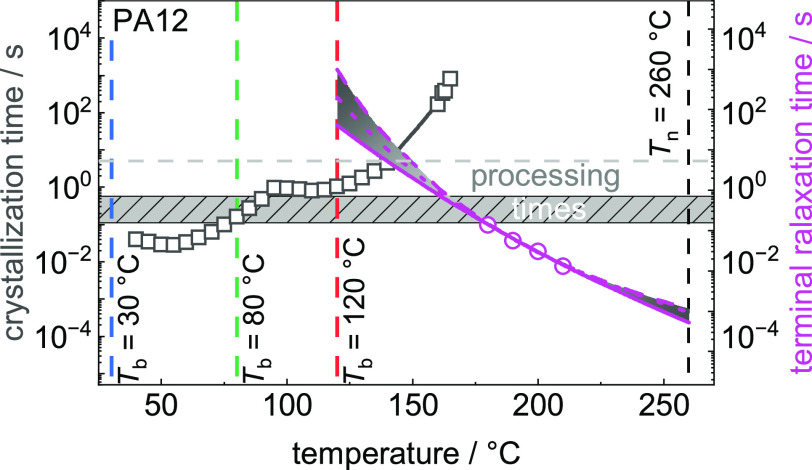

We report the results of a study focusing on the influence of crystallization kinetics and flow behavior on structural inhomogeneities in 3D-printed parts made from polyamide 12 (PA12) and poly(lactic acid) (PLA) by dynamic mechanical analysis (DMA), differential scanning calorimetry (DSC), fast scanning calorimetry (FSC), and wide-angle X-ray diffraction (WAXD). Temperature-dependent WAXD measurements on the neat PLA filament reveal that PLA forms a single orthorhombic α phase during slow cooling and subsequent 2nd heating. The PA12 filament shows a well pronounced polymorphism with a reversible solid–solid phase transition between the (pseudo)hexagonal γ phase near room temperature and the monoclinic α′ phase above the Brill transition temperature *T*_B_ = 140 °C. The influence of the print bed temperature *T*_b_ on structure formation, polymorphic state, and degree of crystallinity χ_c_ of the 3D-printed parts is investigated by height and depth-dependent WAXD scans and compared with that of 3D-printed single layers, used as a reference. It is found that the heat transferred from successive layers has a strong influence on the polymorphic state of PA12 since a superimposed mixture of γ and α phases is present in the 3D-printed parts. In the case of PLA, a single α phase is formed. The print bed temperature has, in comparison to PA12, a major influence on the degree of crystallinity χ_c_ and thus the homogeneity of the 3D-printed parts, especially close to the print bed. By comparing the obtained results from WAXD, DMA, DSC, and FSC measurements with relevant printing times, guidelines for 3D-printed parts with a homogeneous structure are derived.

## Introduction

Additive manufacturing (AM) is commonly understood as a key factor in changing the economic landscape^1^ in the next few decades since it allows production in a decentralized system and delivery on demand.^2^ Fused filament fabrication (FFF), sometimes also called material extrusion AM (MEAM), is one of the most popular AM methods,^3^ where a thermoplastic polymer- or metal filament is extruded through a heated nozzle on a print bed layer by layer. Crystallization in components produced by FFF occurs in a specific manner since heat is introduced repeatedly by the strands positioned on the already exiting part of the component. In combination with the bad heat conductivity of polymers, this results in complicated, spatially heterogeneous temperature profiles. Typical is that the local temperature in a certain volume element can be cycle-dependent on the chosen printing sequence as well as by the actual temperature in the environment.^4^ In addition, shear forces are introduced in the nozzle as well as during positioning the strand on the existing component surface.^5,6^ Thus, crystallization processes in 3D-printed components are quite complex, and a vast variety of parameters, like print bed *T*_b_, chamber temperature, or printing speed *v*, do affect the internal semicrystalline structure.^3,4,7−10^ Another crucial aspect for getting homogeneous 3D-printed components, which has been extensively studied in recent years, is to avoid interlayer surfaces, i.e., a pronounced welding zone.^4−7,11−13^ In situ characterization experiments for various 3D-printed semicrystalline polymers indicate the occurrence of significant inhomogeneity of degree of crystallinity and crystal orientation within individual layers.^5,6,12,13^ The degree of crystallinity in the bulk zone of each layer is found to be higher as compared to regions near the surface or interface for materials like polycaprolactone (PCL), poly(vinylidene fluoride) (PVDF), or isotactic polypropylene (iPP). Moreover, crystal orientation is observed in regions where extruded strands come in contact with a surface, and relations to the limited weld strength between individual layers are discussed. A major influence on the internal structure of a FFF-printed component is also the print bed temperature *T*_b_ since a significant part of the crystallization process takes place when filament is deposited.^3,7^ Costanzo et al. have shown that the first printed layer of a polyamide 12 (PA12) component is thermally affected by four subsequent printed layers. Only from the fifth layer onward, high temperatures induced by nozzle and melt no longer influence the first printed layer.^3^ Srinivas et al. demonstrated for PLA that a large number of layers between the component surface and print bed cause decreased heat transport in PLA, resulting in a higher degree of crystallinity χ_c_ far away from the print bed.^4^ Other studies have shown that the maximum achievable degree of crystallinity χ_c_ is reached if the temperature remains constantly above the glass transition temperature *T*_g_ during printing.^3,9,10^

PA12 and PLA are from our point of view interesting model systems for comparative studies since the relevant crystallization times at processing relevant temperatures are quite different.^14,15^ PA12 rapidly crystallizes within a second, while PLA shows very slow crystallization, usually taking minutes. Another interesting aspect is that PA12 and PLA are both of polymorphic nature, i.e., different crystalline states can form depending on processing conditions.^16−22^ PA12 is obtainable in four crystalline phases that are the α, α′ (monoclinic unit cell), γ, and γ′ ((pseudo)hexagonal unit cell) phases that can be distinguished by their characteristic *d*-spacing in the wide-angle X-ray diffraction (WAXD) region.^19−22^ Post-mortem studies on 3D-printed PA12 components indicate that the (pseudo)hexagonal γ phase is the preferred phase.^7,8^ Interestingly, Qi et al. have also reported recently a mixed crystal structure with a gradient between the surface and core region of a FFF-printed component. An oriented α phase is observed near the surface of the filament, while an increased fraction of the γ phase is found in the core region.^23^ PLA is a semicrystalline polymer that is achievable in the orthorhombic α-, α′-, and γ phases and the β phase with a frustrated structure.^16−18^ Virgin FFF-printed PLA yields a low crystalline or even amorphous structure resulting in poor mechanical properties. To tune the crystallization behavior of PLA and to yield better interlayer chain diffusion, FFF efforts have been achieved by adding plasticizers and nucleating agents,^24^ or by tailoring the chemical composition of PLA filaments.^11^ Recent literature dealing with the influence of different print bed temperatures reports that PLA components printed at low bed temperatures *T*_b_ crystallizes in the α′ phase,^9,10^ while samples printed at high bed temperatures *T*_b_ (>120 °C) have been found to crystallize in the stable α phase.^9^

A commonly applied criterion for the fluidity of polymeric filaments for FFF is to have a zero shear viscosity η_0_ of about 10^2^ to 10^3^ Pa·s, at relevant printing temperatures close to the nozzle.^25−29^ This criterion gives a reasonable guideline for the successful processing of related polymers but neglects the shear rate dependence of the fluidity of the polymeric melt. Since shear rates  in the range of about 10^2^ to 10^3^ s^–1^ occur in the nozzle,^26,29^ this is a relevant restriction that has been included in more detailed studies.^30,31^ The importance of crystallization, shear viscosity, and an applied printing program for the interlayer/welding strength and the formation of voids in 3D-printed components has also been explicitly considered in special studies.^32−34^

The main goal of this study is to derive rational criteria that should be fulfilled to obtain homogeneous components without internal interfaces and macroscopic voids by FFF. Filaments from PLA and PA12 have been chosen as model systems since these polymorphic materials differ significantly regarding the crystallization rate in the relevant processing temperature range, while their viscosities can be adjusted to be similar. We demonstrate that this results in different requirements regarding the parameters of the 3D-printing process and will end up with recommendations for the optimization of process parameters and material properties.

## Materials and Methods

### Materials

**Figure 1:**
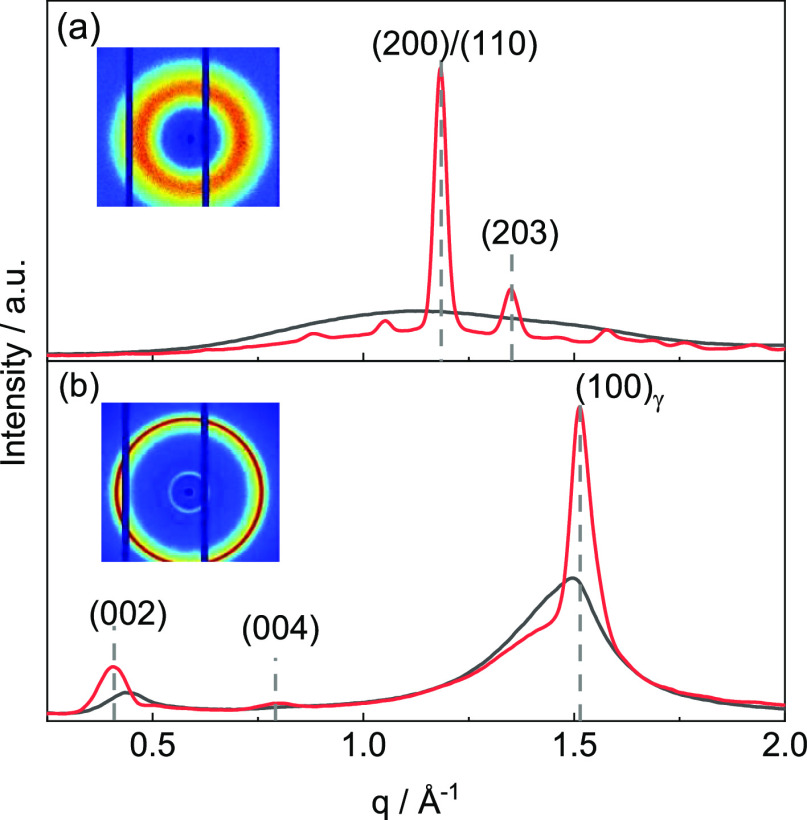
Scattering pattern of (a) PLA and (b) PA12 in the as-received state (black) and measured at 30 °C after slow cooling the relaxed melt (red). Corresponding 2D scattering pattern of the as-received filament are shown in the inset.

PA12 filament was purchased from Fiberlogy, Brzezie, Poland. The as-received filaments are transparent with a diameter of 1.75 mm. The PA12 has a melt flow index of 180 cm^3^/10 min (235 °C/5 kg). Figure 1b shows the 2D scattering pattern along with the azimuthal integrated 1D scattering pattern of the as-received PA12 filament at room temperature as well as the 1D scattering pattern at 30 °C after slow cooling of the relaxed melt. Three major Bragg reflections corresponding to the γ phase (hexagonal unit cell) indexed as 002, 004, and 100 can be seen at scattering vector *q* values of *q*_002_ = 0.49 Å^–1^, *q*_004_ = 0.79 Å^–1^, and *q*_100_ = 1.49 Å^–1^, respectively. The corresponding *d*-spacing (*d* = 2π/*q*) are *d*_002_ = 12.82 Å, *d*_004_ = 7.95 Å, and *d*_100_ = 4.22 Å, respectively. The degree of crystallinity χ_c_ obtained from the areas of the crystalline peaks *A*_*hkl*_ and the amorphous halo *A*_amo_, is about 23%. Weak anisotropy in the intensity distribution of the 2D pattern is indicated by the local intensity maxima of the 002 reflection. The PA12 filament exhibits, besides the hexagonal γ phase at room temperature, a high temperature α′ phase (monoclinic unit cell) above the Brill transition temperature *T*_B_ = 140 °C, as shown in the results from temperature-dependent WAXD measurements in the Supporting Information (cf. Figure S1).

PLA filament (Renkforce RF-4511200) was purchased from Conrad Electronic SE, Hirschau, Germany. The as-received filament is transparent and its diameter is 1.75 mm. This grade contains >97.9% PLA (CAS no. 26100-51-6), 2% styrene/butadiene copolymer (CAS no. 9003-55-8), and <0.1% other additives. The mass averaged molecular weight *M*_w_ and polydispersity index (PI) were measured by gel permeation chromatography (Omnisec, Malvern Panalytical, United Kingdom) against polystyrene standards, to be 204 and 1.4 kg/mol, respectively. Figure 1a presents the 2D scattering pattern and the azimuthal integrated 1D scattering pattern of the PLA filament in the as-received state, along with the 1D scattering pattern at 30 °C after slow cooling the relaxed melt. The absence of Bragg reflections in the as-received filament reveals that the PLA filament is in a fully amorphous state. After slow cooling, the relaxed melt PLA is present in the orthorhombic α phase, identified by two major Bragg reflections in the WAXD region along with further weak Bragg reflections. The major Bragg reflections are indexed as 200/110 and 203 occurring at *q*_200/110_ = 1.18 Å^–1^ and *q*_203_ = 1.35 Å^–1^. The corresponding *d*-spacing values are *d*_200/110_ = 5.32 Å and *d*_203_ = 4.65 Å. Temperature-dependent WAXD measurements reveal that PLA forms a single orthorhombic α phase during stepwise cooling the melt and subsequent 2nd heating (cf. Figure S4).

### Differential Scanning Calorimetry

The crystallization kinetics of PA12 and PLA under nonisothermal and isothermal conditions were investigated using a heat-flux DSC 1 (Mettler-Toledo, Switzerland), which is equipped with a FRS 5 sensor and connected to a Huber Intracooler TC100. The purge gas was nitrogen, with a flow rate of 60 mL/min. Sealed aluminum pans with a volume of 40 μL were used, and the sample mass was about 4 mg. The first heating runs to determine the initial crystalline structure of PLA and PA12 filaments were performed at a heating rate of 20 K/min. More details of the temperature–time protocol will be discussed in the main text.

### Fast Scanning Calorimetry

A Flash DSC from Mettler-Toledo, Switzerland, equipped with a chip-sensor (MultiSTAR UFS 1, Xensor-Integration, The Netherlands) and a Huber intracooler TC100 was used for FSC measurements. The purge gas was nitrogen, with a flow rate of 30 mL/min. A tiny particle with a sample mass of about 50–100 ng was placed on the FSC sensor. A good and stable thermal contact between the sample and the sensor was obtained after several melting-crystallization cycles. In nonisothermal measurements, cooling rates were varied between 10 and 5000 K/s, and an optimum rate of 1000 K/s was applied for subsequent heating. Furthermore, isothermal crystallization of PA12 in the temperature range between 40 and 140 °C was investigated. The mass of FSC samples was estimated by the absolute, measured heat capacity difference at the glass transition temperature *T*_g_ of a fully amorphous sample, with the corresponding mass-normalized values available in the literature.^35,36^

### Dynamic Mechanical Analysis

Dynamic mechanical measurements were performed on an AntonPaar MCR502 rheometer in plate–plate geometry (plate diameter of 8 mm). Samples were prepared by pressing plates in a small mold using a heated hydraulic press (Collin) for approximately 4 min at 190 °C (PLA) and 260 °C (PA12) with a pressure of 2 bar to form a disc with a diameter of 24.5 mm and a height of ≈1 mm. Subsequently, a specimen with a diameter of 8 mm was punched out of the disc. Before being measured, the specimen was placed between the plates in a preheated chamber (190 °C PLA and 210 °C PA12) and homogenized with a shear deformation of 1%. After homogenization, the gap between the plates was reduced to ≈0.7 mm. Isothermal frequency sweeps were measured during cooling between 210 and 150 °C for PLA and between 210 and 180 °C for PA12 in steps of 10 K. Frequency sweeps were performed in a frequency range 0.01–100 rad/s with a shear strain of 2% at constant temperature. Master curves were constructed with TA Orchestrator software.

### 3D-Printing

FFF parts were manufactured on a *A*4*v*4 printer by 3ntr with a multimaterial high temperature hotend print head (with three nozzles) with a diameter of 0.4 mm on a heated carbon fiber reinforced polymer (CFRP)-built plate. Using the software package Simplify3D, a CAD file (constructed in 3ds CATIA V5) was sliced and exported as a G-Code file. After compilation, the G-Code was sent to the 3ntr Print Server, where printing parameters were set. Table 1 presents nozzle temperature *T*_n_, print bed temperature *T*_b_, printing speed *v*, width of layer *w*_l_, height of layer *z*_l_, extrusion multiplier *E*_m_, total height of component *z*, and height of the filled part *z*_f_, used for printing of PA12 and PLA components. Typical repetition times in-plane *t*_r,ip_ and out-of-plane *t*_r,op_ have been determined directly from the G-code. Average *t*_r,ip_ values are 1.8 to 4.9 s, while *t*_r,op_ is commonly 18 s. These times correspond to the time period needed by the nozzle to reach the neighbored position of a certain volume element within the plane again and in the next plane, respectively.

**Table 1:** Printing Parameters and Dimensions of the 3D-Printed Components

material [—]	*T*_n_ [°C]	*T*_b_ [°C]	*v* [mm/s]	*w*_l_ [mm]	*z*_l_ [mm]	*E*_m_ [%]	*z* [mm]	*z*_f_ [mm]
PA12	260	30, 80, 120	50	0.55	0.30	80	60	20
PLA	190	30, 60, 90	50	0.55	0.30	100	60	20

*E*_m_ was used for optimization of the extrusion flow rate to obtain the best results when depositing the filament.

**Figure 2:**
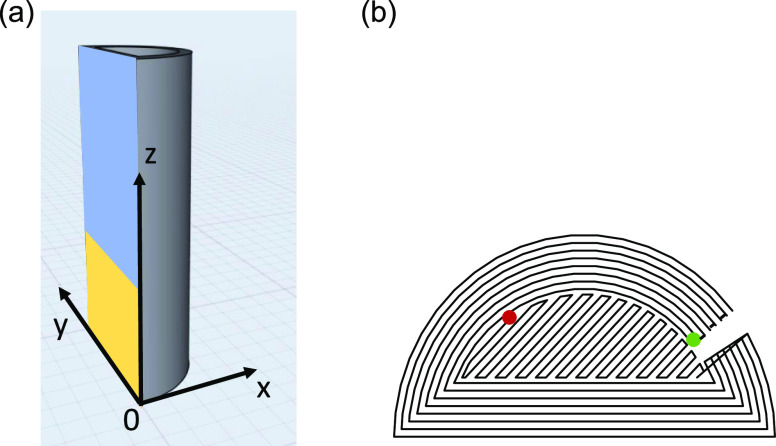
(a) CAD drawing of the chosen model component with a coordinate system. (b) Top view on one layer. Trace of nozzle starting with innermost perimeter (green dot) printing to outermost perimeter, followed by filling stage in angle of ±45°. End point of layer printing is indicated by a red dot.

The dimensions of the 3D-printed half-cylinders are height *z* = 60 mm, diameter *y* = 30 mm, and depth *x* = 15 mm. The printed part consists of a filled core region up to a height *z* of 20 mm, while the height *z* of the hollow part is 40 mm with a wall thickness of 2 mm (Figure 2a). The printing of the components was divided into two stages: the printing of the perimeter and the filling stage. In total, eight perimeters were printed from the innermost to the outermost perimeter, followed by the filling stage with an angle of ±45° (Figure 2b). In order to ensure sufficient adhesion between the extruded filament and the print bed, a layer of painter’s tape with a thin film of water diluted wood glue (1:1 ratio) was attached to the print bed. The *Z*-offset between the nozzle and print bed was 2 mm. The single layers of PLA and PA12 were also printed with the same printing parameters as used for printing the respective components, i.e., an analogous waiting period was applied before the component was removed from the bed.

### Microtomy

**Figure 3:**
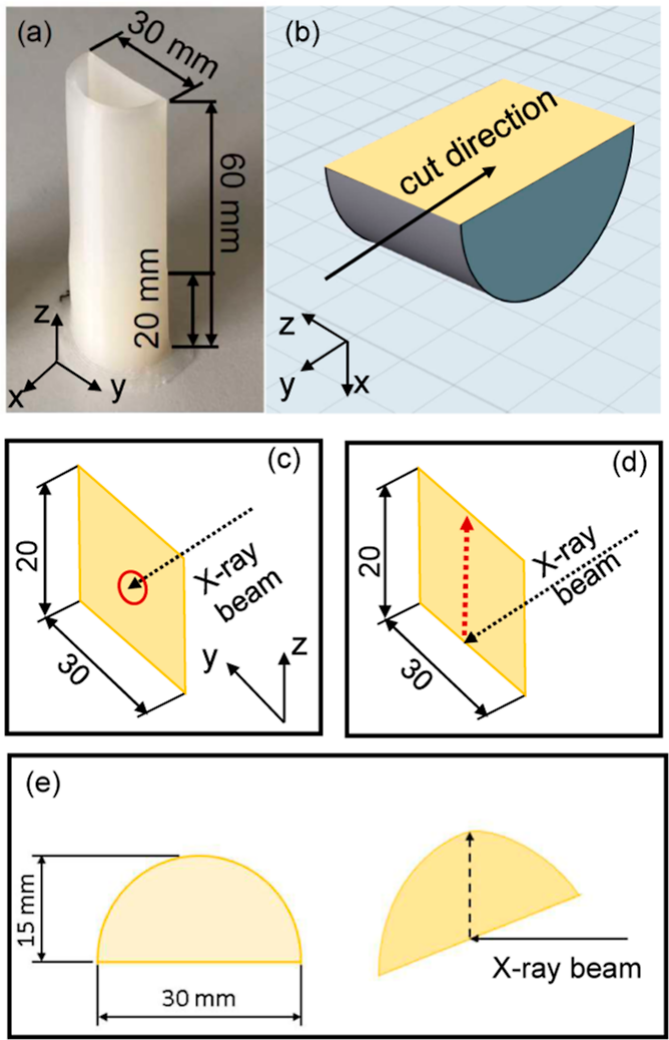
(a) 3D-printed component consisting of a lower filled part (*z* ≤ 20 mm) and an upper hollow part (20 mm ≤ *z* ≤ 60 mm); (b) CAD model of the filled part of 3D-printed components for microtomy; and (c) microtomed thin section for depth-dependent measurement. Red circle shows the point of measurement at *y* = 15 mm and *z* = 10 mm (10 mm from bottom); (d) thin section used for height-dependent measurements along the red dashed line (fixed *x* = 2.5 mm and *y* = 15 mm) from *z* = 0 mm to *z* = 20 mm; and (e) single layer and direction of scanning to study the depth dependence at from *x* = 0 to 15 mm.

In a first step, the 3D-printed components were separated into two parts: the filled part (0 ≤ *z* ≤ 20 mm) and the hollow part (20 ≤ *z* ≤ 60 mm) (Figure 3a). The filled part was clamped in the holding device of the microtome with the flat side of the half cylinder, aligned parallel to the knife of the microtome (Figure 3b). Subsequently, thin sections were then gradually prepared by a microtome with a thickness of 100 μm.

### X-ray Diffraction

X-ray diffraction experiments were performed in transmission mode using a SAXSLAB laboratory setup (Retro-F) equipped with an AXO microfocus X-ray source with an AXO multilayer X-ray optic (ASTIX) as monochromator for Cu K_α_ radiation (λ = 1.54 Å). A DECTRIS PILATUS3 R 300 K detector was used to record the 2D scattering pattern. The sample–detector distance was about 10 cm. Calibration of the sample–detector distance was performed using a silver behenate standard. A twin pinhole system was used for the measurements with an aperture size of 0.9 × 0.9 and 0.4 × 0.4 mm^2^, respectively. The resulting beam size at the sample position was therefore slightly larger than that of a single 3D-printed layer. Spatial heterogeneities within individual layers or strands—as reported in other studies^6,13^—cannot be resolved here. Average values for crystallinity and orientation were observed.

Temperature-dependent WAXD measurements on the as-received filament samples were performed using a Linkam hot stage. The samples were placed in a small hole in an aluminum disc with a diameter of 2 mm. The temperature range for PLA was *T* = 30 to 190 °C, while for PA12, the measured temperature range was from *T* = 30 to 260 °C in steps of 10 K. The heating and cooling rate between temperatures was set as ±10 K/min. The measurement time at each temperature was 5 min. The first heating run was performed to erase the thermal history of the filaments.

WAXD measurements on the thin sections (about 100 μm thick) were performed at room temperature. To study the depth dependence, thin sections taken from different depths *x* were mounted on the Linkam stage so that the center of the section (*z* = 10 mm and *y* = 15 mm) was located in the beam (Figure 3c). In order to investigate structural heterogeneities in 3D-printed components depending on the distance to the bed, height-dependent scans were performed on thin sections at room temperature (Figure 3d). The measurements were performed at a fixed *x* = 2.5 mm and *y* = 15 mm position over the entire height of the filled part from *z* = 0 to 20 mm, with a step size of 0.6 mm. To investigate the influence of the print bed temperature *T*_b_ on a single 3D-printed layer, scans along the *x* direction (depth) were performed from *x* = 0 to 15 mm with a step size of 0.75 mm, as shown in Figure 3e.

Data evaluation and analysis were performed using OriginLab 2019b. In order to fit the WAXD data, a Gaussian–Lorentz crossfit function was applied to determine the area of the amorphous halo *A*_amo_ and Bragg reflections *A*_*hkl*_ (cf. Figures S2, S5, and S7). The regions of integration for PLA and PA12 were 0.5 Å^–1^ ≤ *q* ≤ 2.0 Å^–1^ and 1.0 Å^–1^ ≤ *q* ≤ 2.0 Å^–1^, respectively. The degree of crystallinity χ_c_ was calculated from
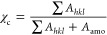
1where Σ*A*_*hkl*_ is the total peak area of all relevant Bragg reflections, while *A*_amo_ is the area of the amorphous halo.

## Results

### Crystallization Kinetics

**Figure 4:**
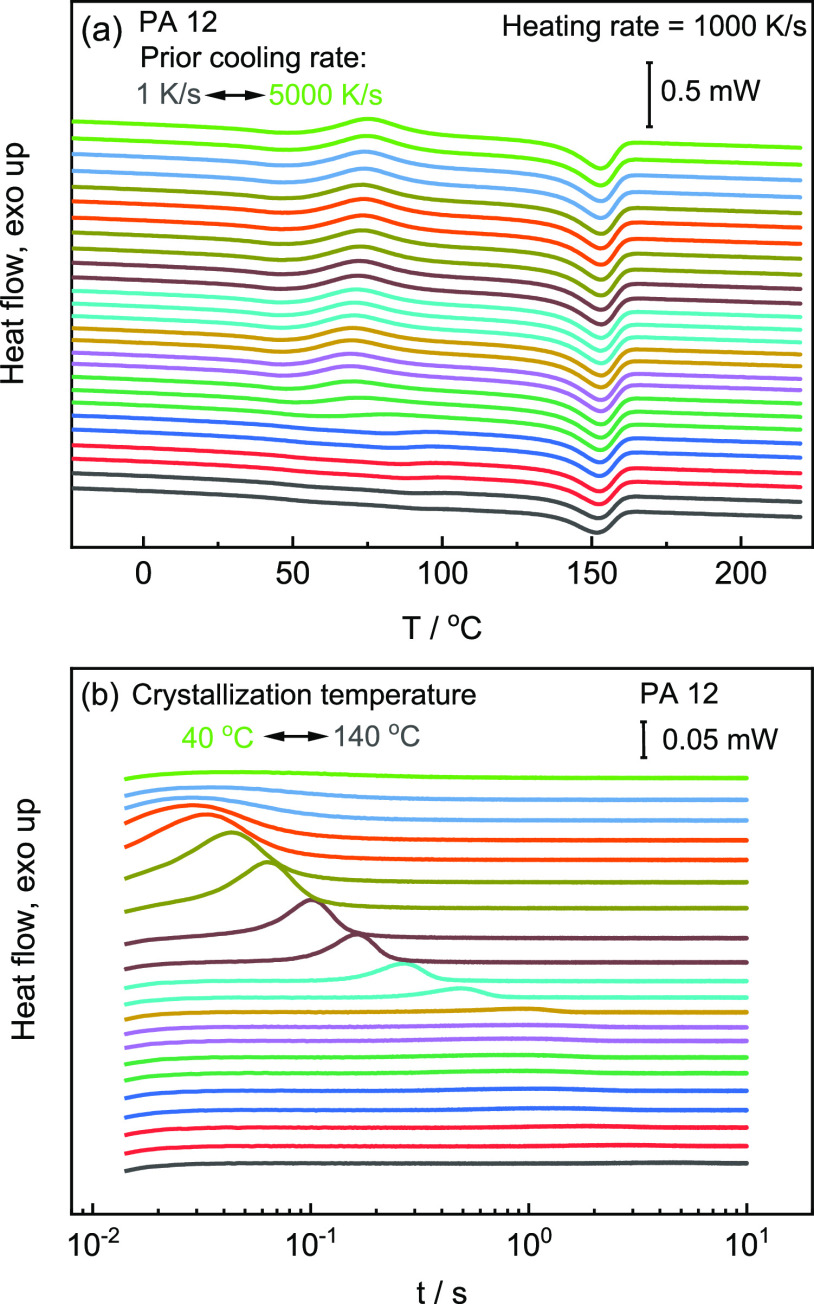
(a) FSC heating scans of PA12 with a heating rate of 1000 K/s after cooling with different rates from 1 to 5000 K/s. (b) Isothermal FSC scans at crystallization temperatures between 40 and 140 °C.

In Figure 4a, FSC heating scans of PA12, after prior cooling from 220 to −60 °C at rates varying from 1 to 5000 K/s, are plotted. The enthalpy of crystallization during prior cooling is estimated through (1) integrating the curves in the temperature range from about 55 to 200 °C and then (2) dividing the obtained value by the heating rate applied (1000 K/s). A straight line is applied as the baseline. In Figure 4b, the heat flow of PA12 during isothermal crystallization is shown. The time that heat flow reaches a maximum is determined as the peak-time of crystallization.

**Figure 5:**
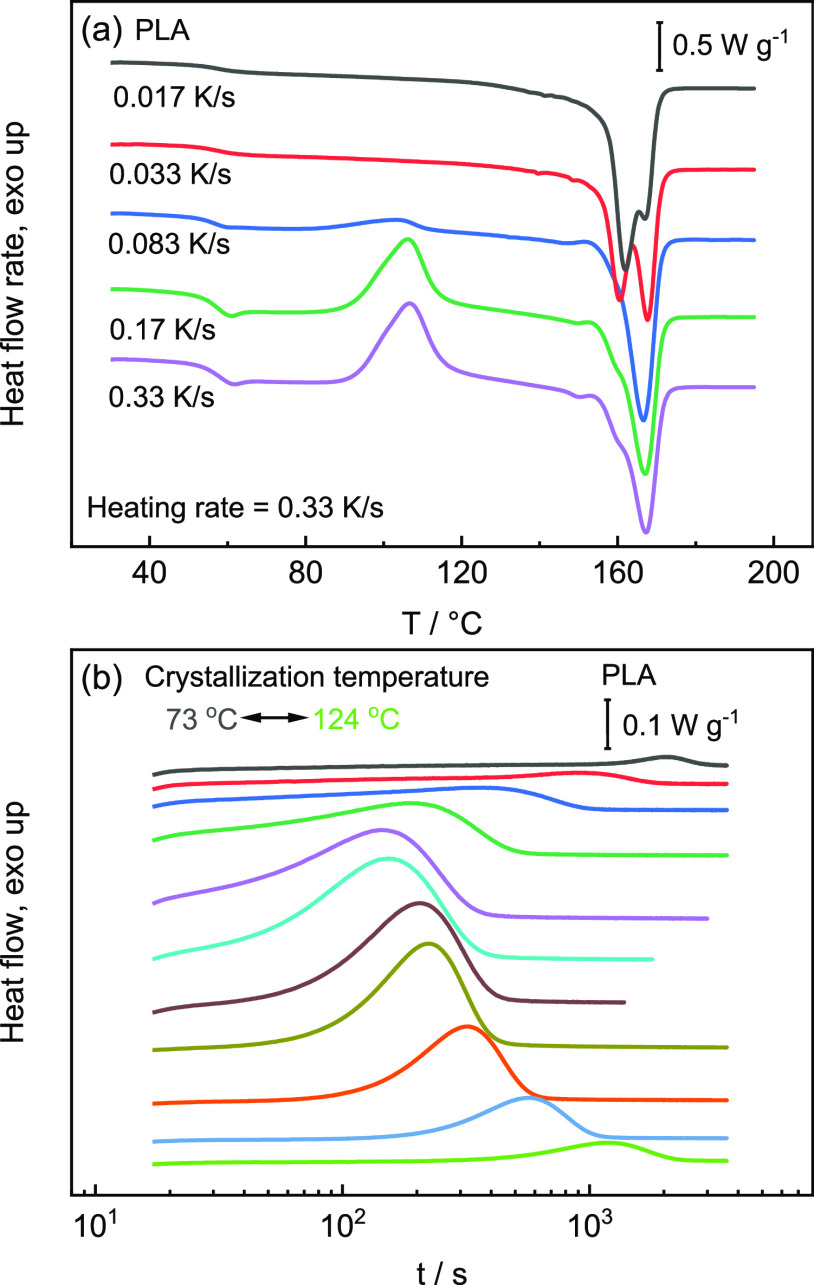
(a) DSC heating scans for PLA at a heating rate of 0.33 K/s after cooling with rates between 0.017 and 0.33 K/s. (b) Isothermal DSC scans at temperatures between 73 and 124 °C.

In Figure 5a, DSC heating scans of PLA, after prior cooling from 200 to 25 °C at rates varying between 0.017 and 0.33 K/s, are plotted. Similar to PA12, the enthalpy of crystallization during prior cooling is estimated through (1) integrating the curves in the temperature range from around 70 to 190 °C and then (2) dividing the obtained values by the heating rate applied (0.33 K/s). Also here, a straight line is used as a baseline. Figure 5b shows the heat flow as a function of time at temperatures between 73 and 124 °C. Similar as in the case of PA12, the time that heat flow reaches its maximum is determined as the peak-time of crystallization.

**Figure 6:**
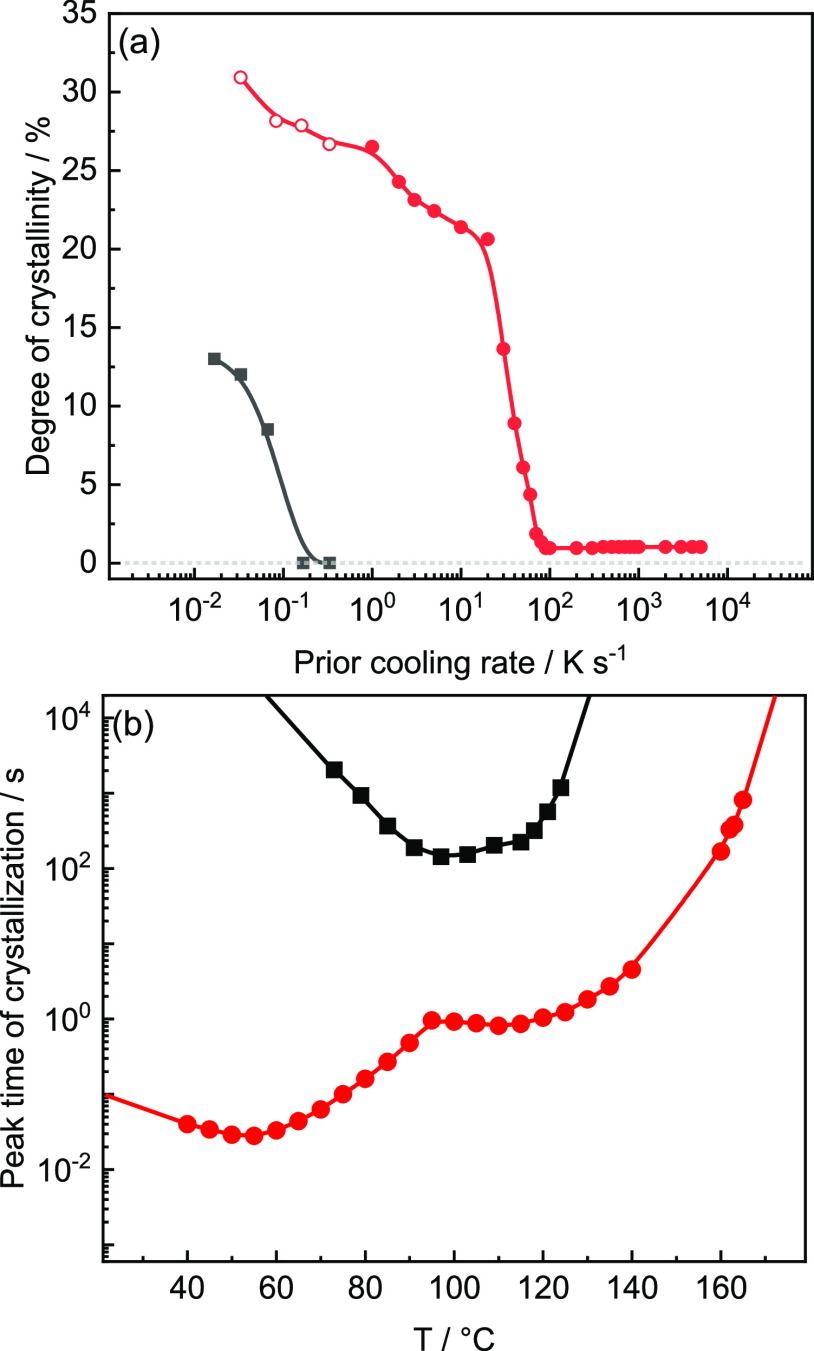
(a) Enthalpy-based crystallinity of PLA (black) and PA12 (red) as a function of cooling rate from nonisothermal crystallization. Red open circles are the degree of crystallinity, determined in nonisothermal DSC measurements. Values of Δ*H*_*m*,100_ to determine the degree of crystallinity are 104 J/g (PLA)^37^ and 4.184 × 50 J/g (PA12)^39^ and (b) peak time of crystallization as a function of temperature in the temperature range from 73 to 124 °C for PLA and between 40 and 140 °C for PA12.

The nonisothermal crystallization kinetics of PLA and PA12 are evaluated through the crystallinity as a function of the prior cooling rate, as shown in Figure 6a, according to the procedure reported in the literature.^37,38^ When the cooling rate is above 0.17 K/s, negligible crystallization occurs during the cooling of PLA, while for PA12, crystallization is absent during cooling faster than 100 K/s. Then, critical cooling rates to suppress crystallization are determined as 0.17 and 100 K/s for PLA and PA12, respectively. Comparing both, it can be seen that the maximum crystallization rate of PA12 is much higher than in the case of PLA. Isothermal crystallization kinetics of PLA and PA12 are evaluated through the peak-time of crystallization as a function of crystallization temperature, as shown in Figure 6b.

The peak-time of crystallization represents the crystallization rate at a particular crystallization temperature. PA12 shows a bimodal dependence of the crystallization time on temperature, with minima at about 5 and 115 °C, respectively. Unlike PA12, a distinct bimodal dependence cannot be seen in the crystallization times as a function of temperature for PLA. Only a weak kink at around 110 °C points to the formation of α′- and α-crystals at low and higher temperatures with different kinetics, respectively. Comparing PLA and PA12 proposes that the crystallization times under isothermal conditions for PA12 are commonly much shorter than those of PLA.

### Flow Behavior in the Molten State

**Figure 7:**
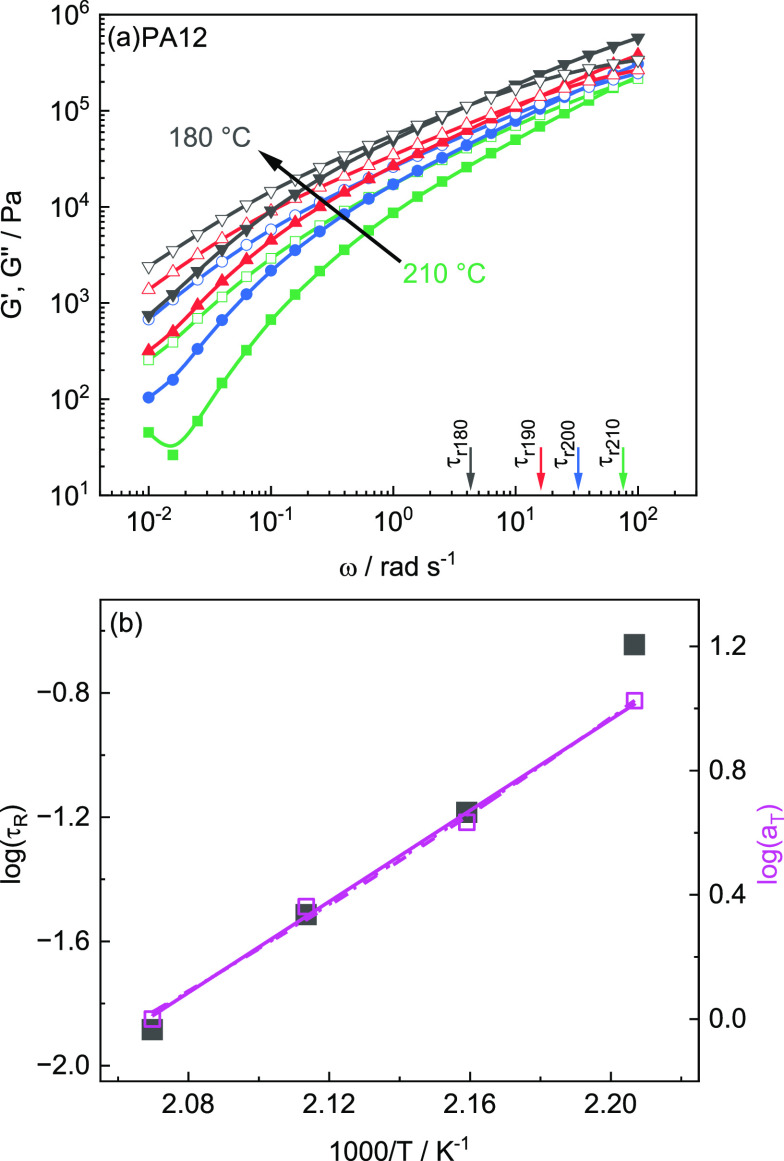
(a) Isothermal sweeps for storage modulus *G*′ (full symbols) and loss modulus *G*″ (open symbols) of PA12 as a function of angular frequency ω for temperatures between 210 and 180 °C. Terminal relaxation time τ_R_ at crossover modulus *G*_c_ of the respective temperature indexed with a colored arrow on the ω-axis; (b) Arrhenius plot of log τ_R_ (full symbols) and log *a*_T_ (open symbols) vs 1000/*T* for PA12. Solid line is an Arrhenius fit corresponding to an activation energy *E*_A_ = 60.9 kJ/mol for the shift factors *a*_T_. Dashed and dotted lines correspond to a VFTH approximation with fixed *T*_V_ = *T*_g_ – 20 K (*A* = 6.9 × 10^–8^ and *B* = 1006 K) and *T*_V_ = *T*_g_ – 70 K (*A* = 1.9 × 10^–9^ and *B* = 1043 K), respectively. *T*_g_ = 314 K is taken from ref (41).

Isothermally measured scans for the shear storage *G*′ and loss modulus *G*″ as a function of angular frequency ω, *G*′(ω), and *G*″(ω) in the flow transition region of PA12 are shown in Figure 7a. The intercepts of *G*′(ω) and *G*″(ω) can be associated with the transition from the rubbery to the liquid-like state. Hence, temperature-dependent terminal relaxation times τ_R_ can be estimated from the crossover frequencies ω_C_ according to τ_R_ = 1/ω_C_. The intercept shifts like the entire flow transition range to lower frequencies ω with decreasing temperature since relevant motions slow down and the terminal relaxation time τ_R_ increases. Note that a Newtonian flow behavior is not achieved in the investigated frequency window for PA12 up to 210 °C.

The Arrhenius plot in Figure 7b demonstrates that log(τ_R_) is decreasing nearly linearly with reciprocal temperature 1000/*T* in the investigated temperature range. An activation energy of *E*_A_ = 74.9 kJ/mol is taken from an Arrhenius fit according to τ_R_ = *C*·exp(*E*_A_/*RT*). The shift factors log(*a*_T_) as obtained from a horizontal master curve construction are also plotted in Figure 7b and given in the Table of the Supporting Information. The activation energy estimated based on the *a*_T_ values is *E*_A_ = 60.9 kJ/mol, which is slightly lower than that obtained from τ_R_. This indicates the existence of experimental uncertainties in the case of data for only four temperatures. An Arrhenius extrapolation of the terminal relaxation times τ_R_ to higher, processing relevant temperatures can be understood as a lower limit for τ_R_ in this region. In general, the temperature dependence of τ_R_ should follow Vogel–Fulcher–Tammann–Hesse (VFTH) behavior^40^
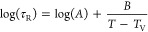
2where *T*_V_ is the Vogel-temperature associated with the temperature where τ_R_ and viscosity η would diverge, *A* is the extrapolation of the relaxation time to an infinite temperature and *B* is related to the curvature. In the case of PA12, the temperature window where the flow behavior can be experimentally investigated between crystallization and degradation temperature is rather limited. Hence, a VFTH fit to the experimental data for τ_R_ and *a*_T_ without additional assumptions is not giving useful results. In order to estimate τ_R_ at processing relevant temperatures based on a VFTH extrapolation, the *T*_V_ values were therefore fixed to *T*_g_ – 70 K and *T*_g_ – 20 K assuming that these are the limits of the interval where *T*_V_ will commonly appear. The results of different extrapolations are given as lines in Figure 7b.

**Figure 8:**
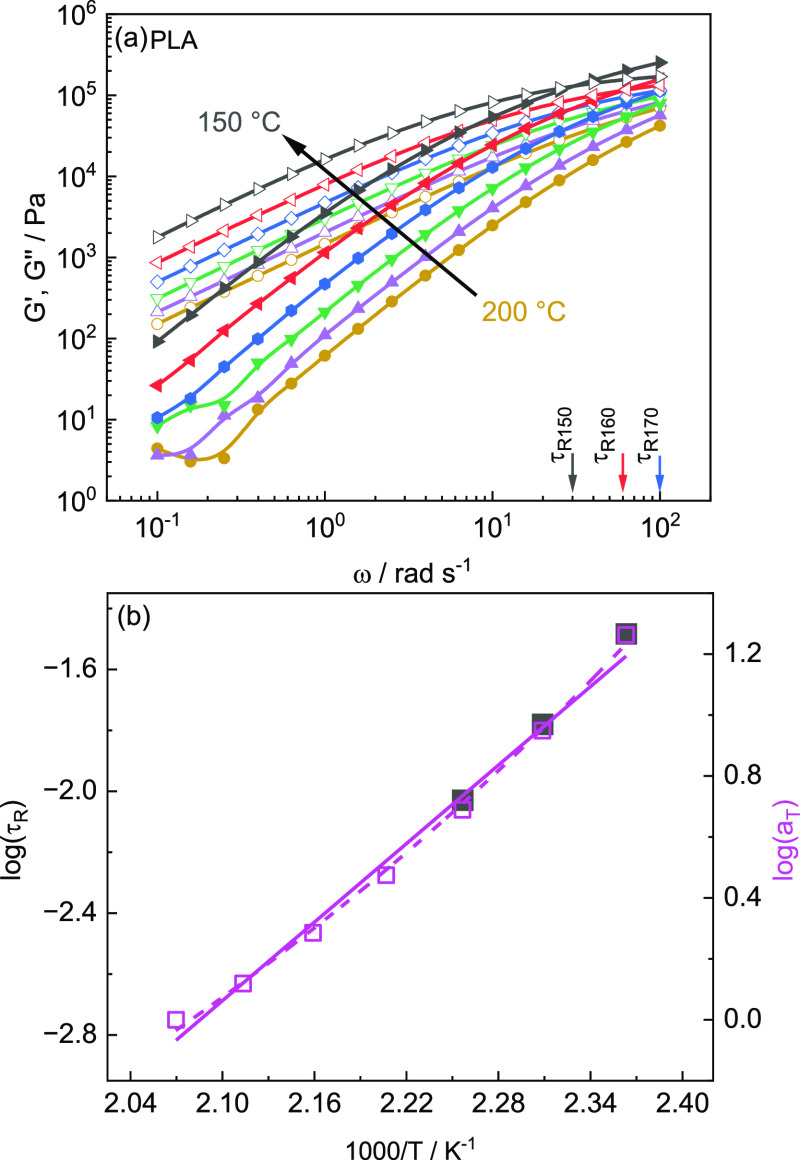
(a) Isothermal sweeps for storage modulus *G*′ (full symbols) and loss modulus *G*″ (open symbols) for PLA as a function of angular frequency ω for temperatures between 200 and 150 °C. Terminal relaxation time τ_R_ of the respective temperature is indexed with colored arrows on the ω-axis. (b) Arrhenius plot of log(τ_R_) (full symbols) and log(*a*_T_) (open symbols) vs 1000/*T* for PLA. Solid line is an Arrhenius fit corresponding to activation energy *E*_A_ = 35.7 kJ/mol for the shift factors *a*_T_. Dashed line represents a VFTH estimation with fixed *T*_V_ = 276 K (*A* = 1.4 × 10^–6^ and *B* = 644 K).

Figure 8a shows *G*′ and *G*″ isotherms for PLA measured at temperatures from 210 to 150 °C. Terminal relaxation times τ_R_ are determined from the intercept *G*′(ω) = *G*″(ω), which are available and listed in the Table of Supporting Information together with temperature-dependent values for the horizontal shift factor *a*_T_ as obtained from a master curve construction. In Figure 8b, the logarithm of shift factors log(*a*_T_) and terminal relaxation times log(τ_R_) is plotted as a function of 1000/*T* together with Arrhenius fits giving activation energies of  = 35.7 kJ/mol and  = 40.5 kJ/mol, respectively. The shift factors *a*_T_ for PLA show in the measured temperature interval a certain curvature, but a free VFTH fit is still not giving reasonable results since the available temperature window between crystallization and degradation is too limited. Hence, a VFTH extrapolation based on the shift factors *a*_T_ for PLA is made with a fixed *T*_V_ in order to estimate τ_R_ at relevant processing temperatures. *T*_V_ = 276 K is chosen here using knowledge about the glass transition temperature *T*_g_ = 330 K,^42^ the Vogel temperature of the α relaxation of PLA repoted to be *T*_V_ = 286 K,^43^ and typical relations between *T*_V_ of α relaxation and flow transition in fully amorphous polymers.^44−46^

### Semi-Crystalline Structure of 3D-Printed Components

#### Polyamide 12

**Figure 9:**
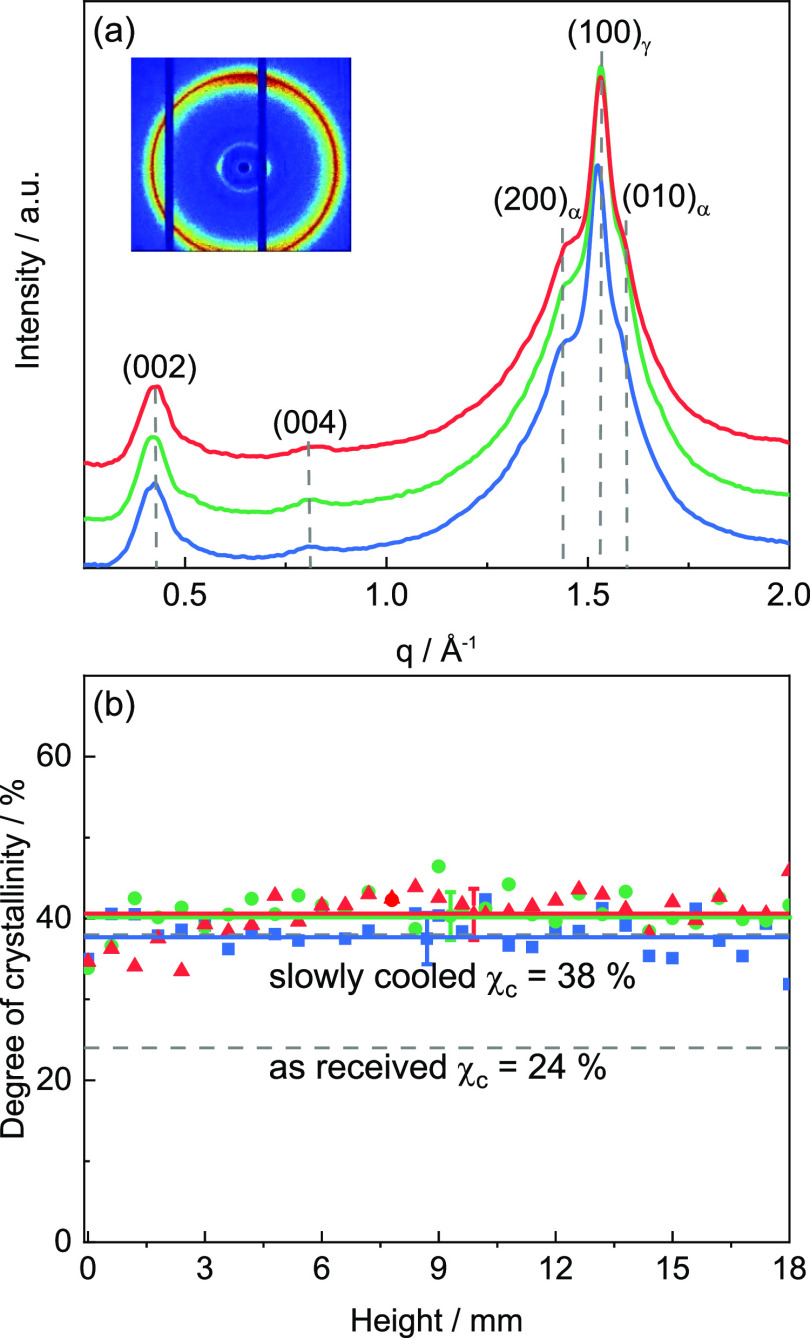
(a) Representative 1D WAXD scattering pattern for PA12 components printed at *T*_b_ = 30 °C (blue), 80 °C (green), and 120 °C (red) at a height of *z* = 0.6 mm (*x* = 2.5 mm and *y* = 15 mm are fixed). Inset shows a representative 2D scattering pattern (*T*_b_ = 30 °C) exhibiting an anisotropic intensity distribution. (b) Degree of crystallinity χ_c_ as a function of height *z*. The degree of crystallinity χ_c_ of the as-received and slowly cooled PA12 from temperature-dependent WAXD scans is indicated by gray dashed lines. Error bars correspond to the standard derivation of mean values from height-dependent measurements.

Height-dependent WAXD scans are performed on thin sections microtomed in a depth of 2.5 mm from the filled part of the components and scanned from the bottom *z* = 0 to 20 mm in steps of 0.6 mm in the middle of the thin section (*y* = 15 mm). Figure 9a shows a comparison of representative 1D scattering patterns measured at a height of 0.6 mm for PA12 components printed at print bed temperatures *T*_b_ of (a) 30, (b) 80, and (c) 120 °C. The WAXD pattern shows for all bed temperatures a characteristic reflection at low *q* values assigned to the (002) plane at *q*_002_ = 0.42 Å^–1^ and *q*_004_ = 0.8 Å^–1^. The reflection corresponds to layered H-bonds along the chain axis, as typically observed for polyamides.^47−50^ In the WAXD range, the pattern for 3D-printed PA12 parts shows one strong Bragg reflection, corresponding to the (100) plane of the (pseudo)hexagonal γ phase observed at *q*_100_ = 1.52 Å^–1^. The corresponding *d*-spacing is *d*_100_ = 4.13 Å. Two additional reflections occur as shoulders at *q* = 1.44 Å^–1^ and *q* = 1.59 Å^–1^. The latter reflections can be attributed to the (200) and (010) planes of the α phase, respectively.^51^

A comparison with the 1D scattering pattern of slowly cooled PA12 (Figure SI1) reveals that the positions of the (200) planes almost match. However, the *q*_010_ value for the 3D-printed component does not agree with that of the (010) plane for the α′-phase, which suggests the appearance of the α phase. Hiramatsu et al. have reported that a mixture of γ′- and α phase is formed by drawing the γ phase of PA12 above 70 °C.^51^ The 1D pattern observed in this case are in approximate agreement with those for the investigated 3D-printed component in Figure 9a.^51^ This suggests that a mixed structure containing γ and α phases occurs in the investigated 3D-printed components made from PA12.

Structural parameters, including the α to γ ratio *R*_γ/α_, are determined by an approximation of the diffraction pattern based on the fit results. The values obtained at a height of *z* = 0.6 mm for different print bed temperatures *T*_b_ are given in Table 2. The values for the coherence length of the γ phase *L*_100_ for *T*_b_ = 30 °C of about 12.3 nm are in good agreement with those found in temperature-dependent measurements, while *L*_100_ is increasing to 12.7 and 13.2 nm for higher print bed temperatures *T*_b_ (80 and 120 °C), indicating the occurrence of slightly larger crystalline domains. Considering the γ-to-α ratio and the overall degree of crystallinity χ_c,tot_ (36–41%) dependence on *T*_b_, one can conclude that the print bed temperature *T*_b_ has a weak influence on the crystalline state close to the print bed for PA12. In Figure 9b, the overall degree of crystallinity χ_c_ is plotted vs height *z* for print bed temperatures *T*_b_ of 30, 80, and 120 °C (at the same *x* and *y* position). The degree of crystallinity χ_c_ is not only similar near to the bed but also for larger heights *z* approximately the same for all three print bed temperatures *T*_b_ (about 40%). Nevertheless, the average degree of crystallinity χ_c_ far from the print bed is seemingly very few percent higher for the higher *T*_b_ values, even if a scatter in the data is within the experimental error range. Slight differences depending on *T*_b_ can also be seen in the γ-to-α phase ratio. The mean fraction of the α phase at print bed temperatures *T*_b_ of 30 and 80 °C is with 0.43 and 0.44 similar, but a significant increase to 0.52 is found for *T*_b_ = 120 °C.

**Table 2:** Structural Parameters Characterizing the Local Crystalline State of Differently 3D-Printed PA12 Components

		α phase				γ phase				
position	*T*_b_ [°C]	*q*_200_ [Å^–1^]	*q*_010_ [Å^–1^]	*d*_200_ [Å]	*d*_010_ [Å]	*q*_100_ [Å^–1^]	*d*_100_ [Å]	*L*_100_ [Å]	χ_c,tot_ [%]	ratio γ/α [—]
*x* = 2.5 mm, *y* = 15 mm	30	1.43	1.58	4.38	3.97	1.52	4.12	122.7	41	1.09
*z* = 0.6 mm	80	1.44	1.57	4.37	3.99	1.53	4.11	126.9	36	1.35
from height-dependent scans	120	1.44	1.59	4.36	3.95	1.53	4.10	132.6	36	0.57
*x* = 0.1 mm, *y* = 15 mm	30	1.43	1.55	4.39	4.05	1.51	4.16	109.3	36	1.16
*z* = 10 mm	80	1.47	1.58	4.29	3.98	1.53	4.12	108.9	43	1.29
from depth-dependent scans	120	1.44	1.58	4.38	3.97	1.52	4.12	113.6	44	0.86

**Figure 10:**
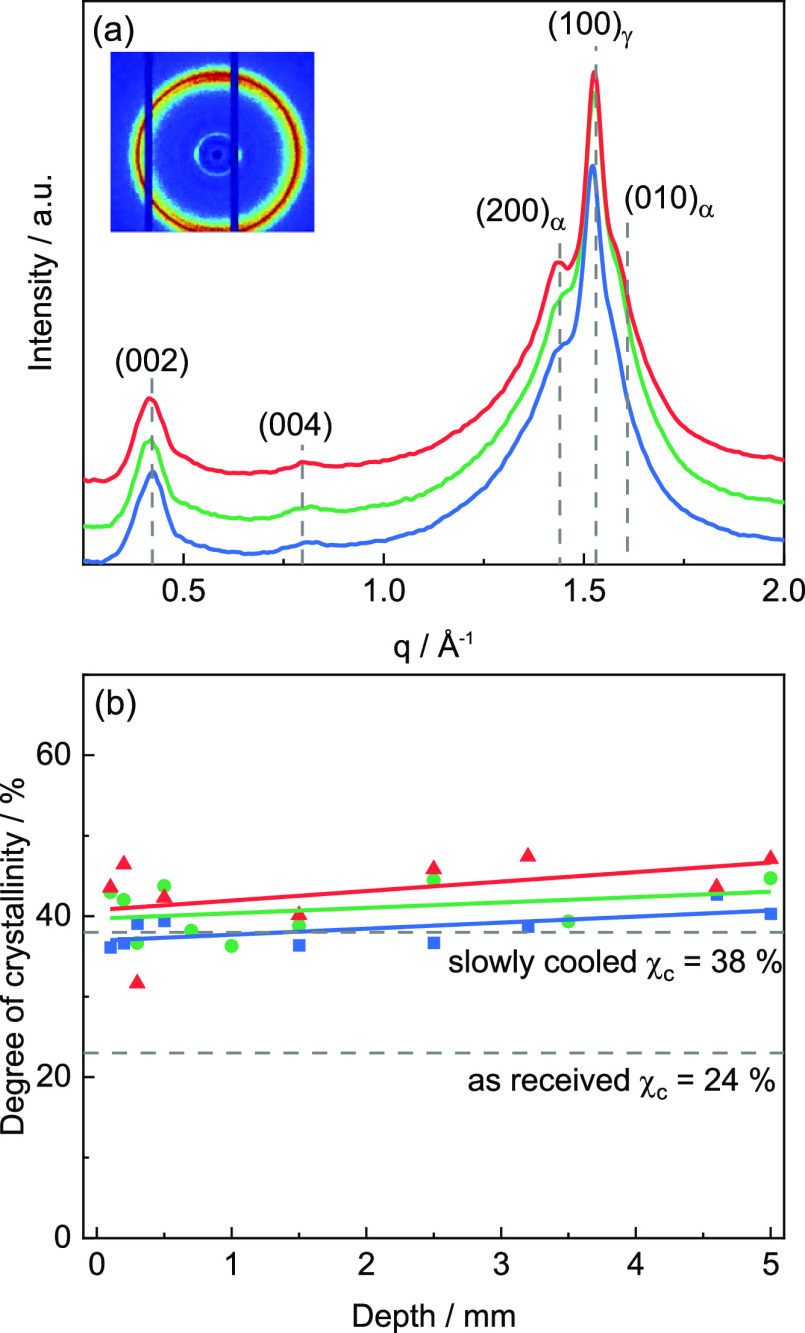
(a) Representative WAXD pattern for PA12 components printed at *T*_b_ = 30 °C (blue), 80 °C (green), and 120 °C (red) at a depth of *x* = 2.5 mm and fixed values for *z* = 10 mm and *y* = 15 mm. Inset shows a representative 2D scattering pattern (*T*_b_ = 30 °C) exhibiting an anisotropic intensity distribution. (b) The degrees of crystallinity as a function of the depth *x* of the 3D-printed PA12 components. Degree of crystallinity χ_*c*_ for slowly cooled and as-received PA12 samples are indicated by dashed lines.

Depth-dependent WAXD studies are performed by measuring microtomed thin sections taken from the filled part of 3D-printed PA12 components (*y* = 15 mm and *z* = 10 mm are fixed) at various depths from *x* = 0.1 to 5.0 mm, approaching the core of the half cylinder. Figure 10a shows a representative 1D scattering pattern for thin sections taken at a depth of *x* = 2.5 mm for PA12 components printed at different print bed temperatures *T*_b_. The 1D WAXD patterns are similar to those obtained from the height-dependent measurements and confirm the coexistence of α and γ phases. A prominent change is, however, that the *q*_(200)_ reflection at 1.43 Å^–1^ is becoming more pronounced with increasing print bed temperatures *T*_b_. The 2D scattering pattern (inset of Figure 10a) basically supports the conclusion that has been derived before from height-dependent measurements. For all investigated depths local intensity maxima at meridional positions are observed for the α phase, while the *q*_100_ reflection representing the γ phase shows basically an isotropic intensity distribution. In Figure 10b, the degree of crystallinity χ_c_ is plotted as a function of depth *x* for PA12 components printed at different print bed temperatures *T*_b_. In average, the degree of crystallinity χ_c_ is slightly higher as compared to the value observed for PA12 samples after slow stepwise cooling (χ_c_ = 38%). Moreover, a certain increase in χ_c_ is observed with increasing depth *x* approaching the core of the component as well as with increasing print bed temperature *T*_b_. In addition, the fraction of α phase is also weakly increasing from 0.47 at *T*_b_ = 30 °C to 0.52 for *T*_b_ = 120 °C. Structural parameters derived from a detailed peak analysis and fitting of the 1D WAXD data for the outer layer (*x* = 0.1 mm) and more close to the core (*x* = 2.5 mm) of the component are compared in Table 2. The reported parameters clearly indicate that the semicrystalline state of the PA12 components at these positions is basically unaffected by the print bed temperature *T*_b_. Only weak changes in the degree of crystallinity χ_c_ and coherence length *L* with the print bed temperature *T*_b_ are observed.

#### Poly(lactic acid)

**Figure 11:**
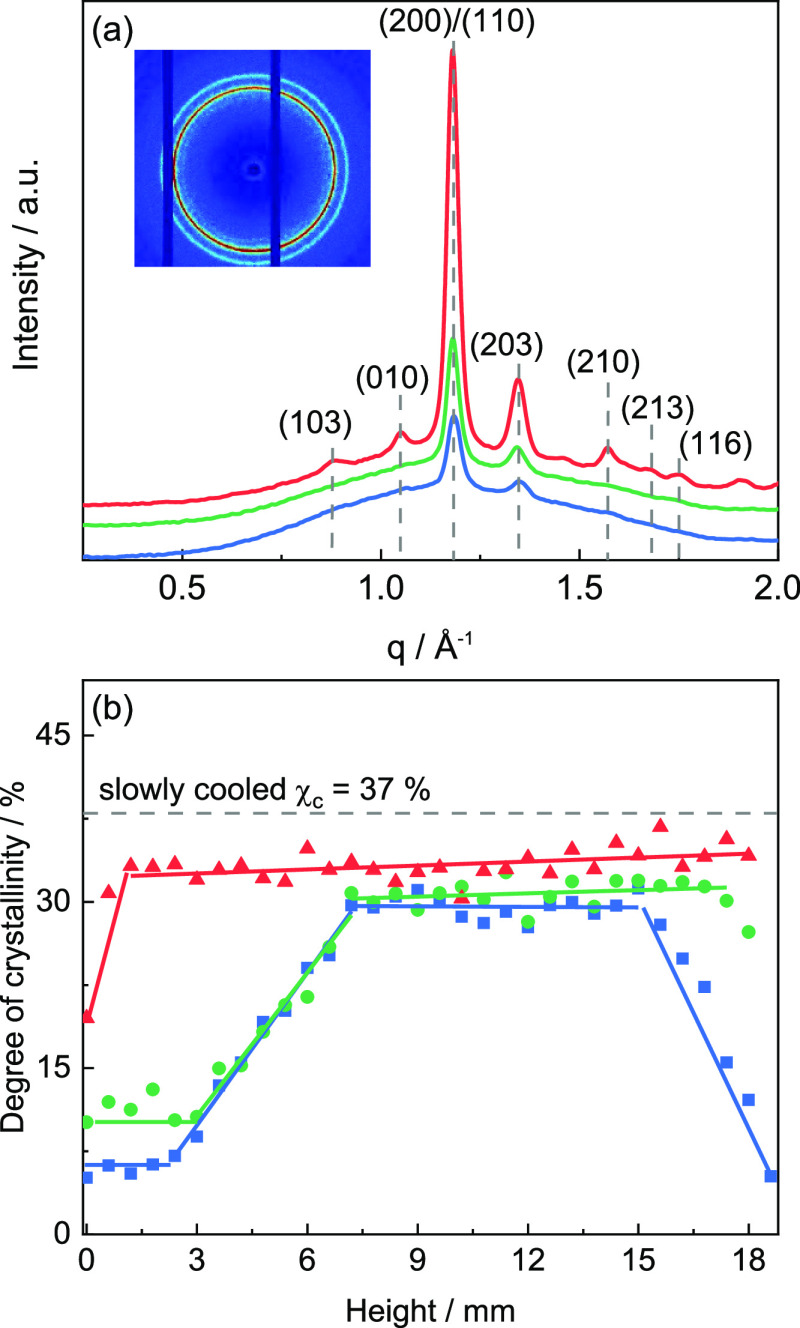
(a) Representative WAXD scattering pattern of PLA components printed at *T*_b_ = 30 °C (blue), 60 °C (green), and 90 °C (red) at a height of *z* = 0.6 mm (*x* = 2.5 mm and *y* = 15 mm are fixed). Inset shows a representative 2D scattering pattern (*T*_b_ = 30 °C) exhibiting an isotropic intensity distribution. (b) Degree of crystallinity χ_c_ as a function of height *z*. Degree of crystallinity χ_c_ of slowly cooled PLA is indicated by a dashed line.

The height-dependence of the local crystalline state is investigated based on thin sections microtomed from PLA components parallel to the *x*–*y* plane. Figure 11a shows a representative scattering pattern for a height of *z* = 0.6 mm measured on sections taken from PLA components printed at three different print bed temperatures *T*_b_. Depth and width coordinates are kept constant (*x* = 2.5 mm and *y* = 15 mm). Two major reflections that are assigned to the (200)/(110) and (203) planes are observed for all print bed temperatures *T*_b_ at *q*_200/110_ = 1.18 Å^–1^ and *q*_203_ = 1.35 Å^–1^. The corresponding *d*-spacings are *d*_200/110_ = Å 5.32 and *d*_203_ = 4.65 Å.

In addition, weaker reflections are commonly found (cf., Figure 11a). Note, however, that these reflections are extremely weak near the printing bed up to a height of *z* = 5 mm for *T*_b_ = 30 and 60 °C. As in the case of stepwise cooling (cf., Figure SI4a) crystallization in 3D-printed PLA occurs always in the orthorhombic α phase with comparable unit cell dimensions (*a* = 10.62 Å, *b* = 6.13 Å, and *c* = 29.00 Å). Table 3 contains structural parameters as obtained from a peak fitting analysis of the corresponding 1D scattering pattern. The results clearly show that the local degree of crystallinity χ_c_ increases strongly with increasing print bed temperature *T*_b_. The 2D scattering pattern shown in the inset of Figure 11a demonstrates that an isotropic intensity distribution is found for 3D-printed PLA components indicating that there is no preferred molecular orientation in conjunction with a relaxation of the initially oriented strand.

**Table 3:** Structural Parameters Characterizing the Local Crystalline State of Differently 3D-Printed PLA Components

position	*T*_b_ [°C]	*q*_200/110_ [Å^–1^]	*q*_203_ [Å^–1^]	*d*_200/110_ [Å]	*d*_203_ [Å]	*L*_200/110_ [Å]	*L*_203_ [Å]	χ_c_ [%]
*x* = 2.5 mm, *y* = 15 mm	30	1.18	1.35	5.31	4.65	171.2	175.0	6
*z* = 0.6 mm	60	1.180	1.35	5.32	4.67	181.1	162.9	12
from height-dependent scans	90	1.18	1.34	5.32	4.67	185.9	150.3	21
*x* = 0.1 mm, *y* = 15 mm	30	1.17	1.34	5.36	4.70	261.8	208.1	32
*z* = 10 mm	60	1.18	1.35	5.31	4.65	168.9	137.8	30
from depth-dependent scans	90	1.19	1.36	5.29	4.64	183.7	157.1	29

Figure 11b presents the degree of crystallinity χ_c_ as a function of the height *z* for PLA components printed at different print bed temperatures *T*_b_. A clear increase in the degree of crystallinity χ_c_ with print bed temperature *T*_b_ is found, especially close to the print bed. For low print bed temperatures (*T*_b_ ≤ 60 °C), the degree of crystallinity χ_c_ increases linearly with height up to *z* = 6 mm and then reaches a saturated value of about 30%. For *T*_b_ = 90 °C, the degree of crystallinity χ_c_ saturates already at *z* ≈ 2 mm and reaches a slightly higher degree of crystallinity χ_c_ of about 34%. The degree of crystallinity χ_c_ near the core of the component seems to depend only slightly on the print bed temperature *T*_b_. In general, the observed trends underline the strong influence of print bed temperature *T*_b_ on the crystalline state of the PLA components. Interestingly, the degree of crystallinity χ_c_ for the PLA components printed at *T*_b_ = 30 °C shows a strong decrease for heights *z* ≥ 15 mm approaching the top of the full part of the component at *z* = 20 mm. This effect is reproducible and much less pronounced for components printed at higher print bed temperatures *T*_b_ (60 and 90 °C). This indicates that the heat transferred from the subsequently printed layers has a significant effect on the degree of crystallinity χ_c_. This applies in particular to components printed at print bed temperatures *T*_b_ below the glass transition temperature *T*_g_ of PLA. Note that this finding is confirmed by WAXD measurements on horizontal sections taken close to the top of the unfilled part (*z* ≈ 20 mm).

**Figure 12:**
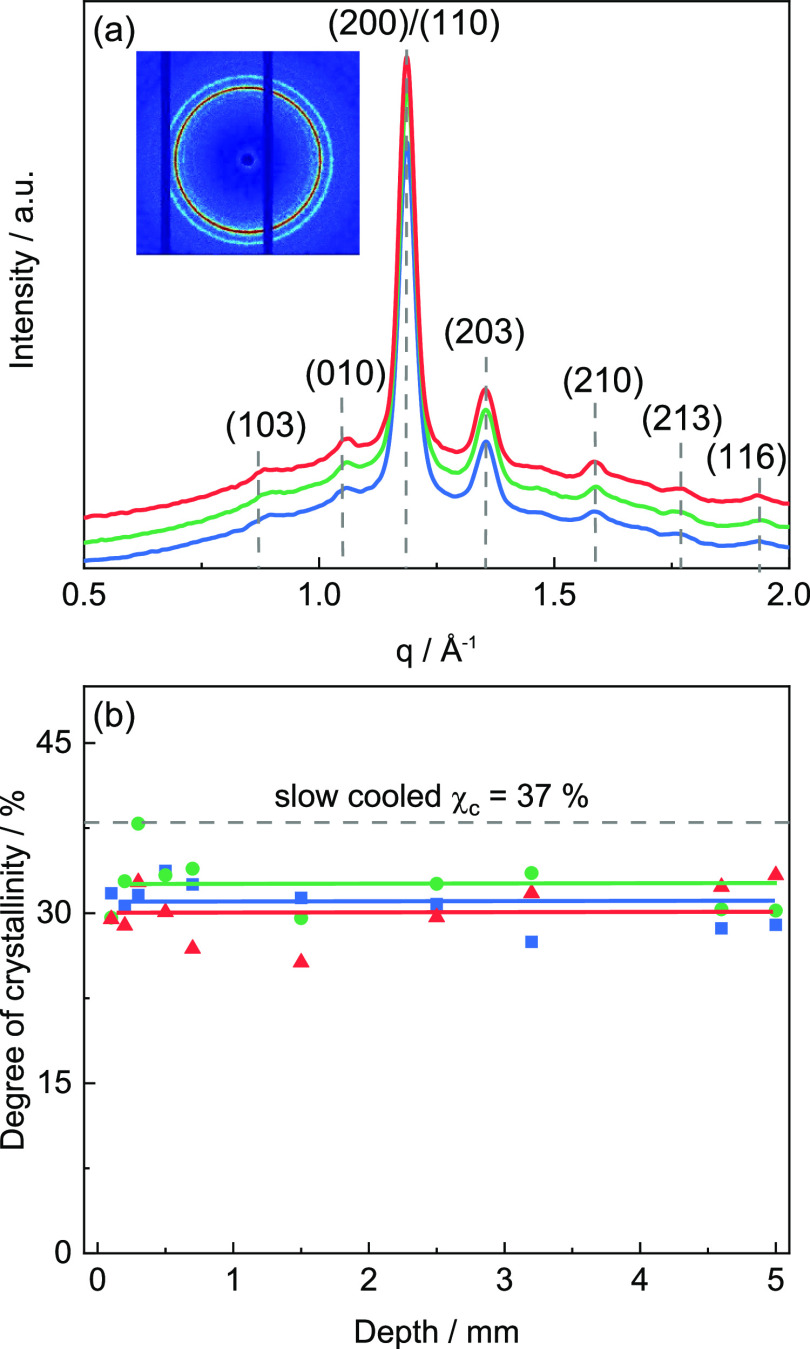
(a) Representative 1D WAXD scattering pattern of PLA components printed at *T*_b_ = 30 °C (blue), 60 °C (green), and 90 °C (red) at a depth of *x* = 2.5 mm (fixed *z* = 10 mm and *y* = 15 mm). Inset shows a representative 2D scattering pattern (*T*_b_ = 30 °C) exhibiting an isotropic intensity distribution. (b) Degree of crystallinity χ_c_ as a function of the depth *x* of 3D-printed PLA components prepared at print bed temperatures *T*_b_ of 30 °C (blue squares), 60 °C (green circles), and 90 °C (red triangles). Degree of crystallinity χ_c_ of slowly cooled PLA is indicated by a dashed line.

Depth-dependent WAXD studies are performed similar to those on PA12 components. Figure 12a shows the representative scattering pattern taken at a depth of *x* = 2.5 mm for PLA components printed at different print bed temperatures. Like in height-dependent WAXD studies, the orthorhombic α phase is commonly observed independent of print bed temperature *T*_b_ and depth *x*. This is evidenced by the characteristic reflections labeled in Figure 12a.

Figure 12b shows data for the degree of crystallinity χ_c_ at print bed temperatures *T*_b_ of 30, 60, and 90 °C as a function of depth *x*. In contrast to the χ_c_ values from the height-dependent scans, the degree of crystallinity χ_c_ remains here constant. It is scattering around 30% nearly, irrespective of print bed temperature *T*_b_ and depth *x*. This indicates that the heat transferred to the print bed or from subsequently printed layers is most relevant to the crystalline state appearing in 3D-printed PLA components. Hence, the position within a layer seems to be less relevant for χ_c_.

The structural parameters from peak fitting are listed in Table 3. The values at print bed temperatures *T*_b_ of 60 and 90 °C are in good agreement with the data from height-dependent scans performed at similar heights (*z* = 2.5 mm vs *z* = 10 mm), while differences are seen for *T*_b_ = 30 °C since the height difference shows a much stronger influence in this case.

### Semi-Crystalline Structure of 3D-Printed Single Layers

**Figure 13:**
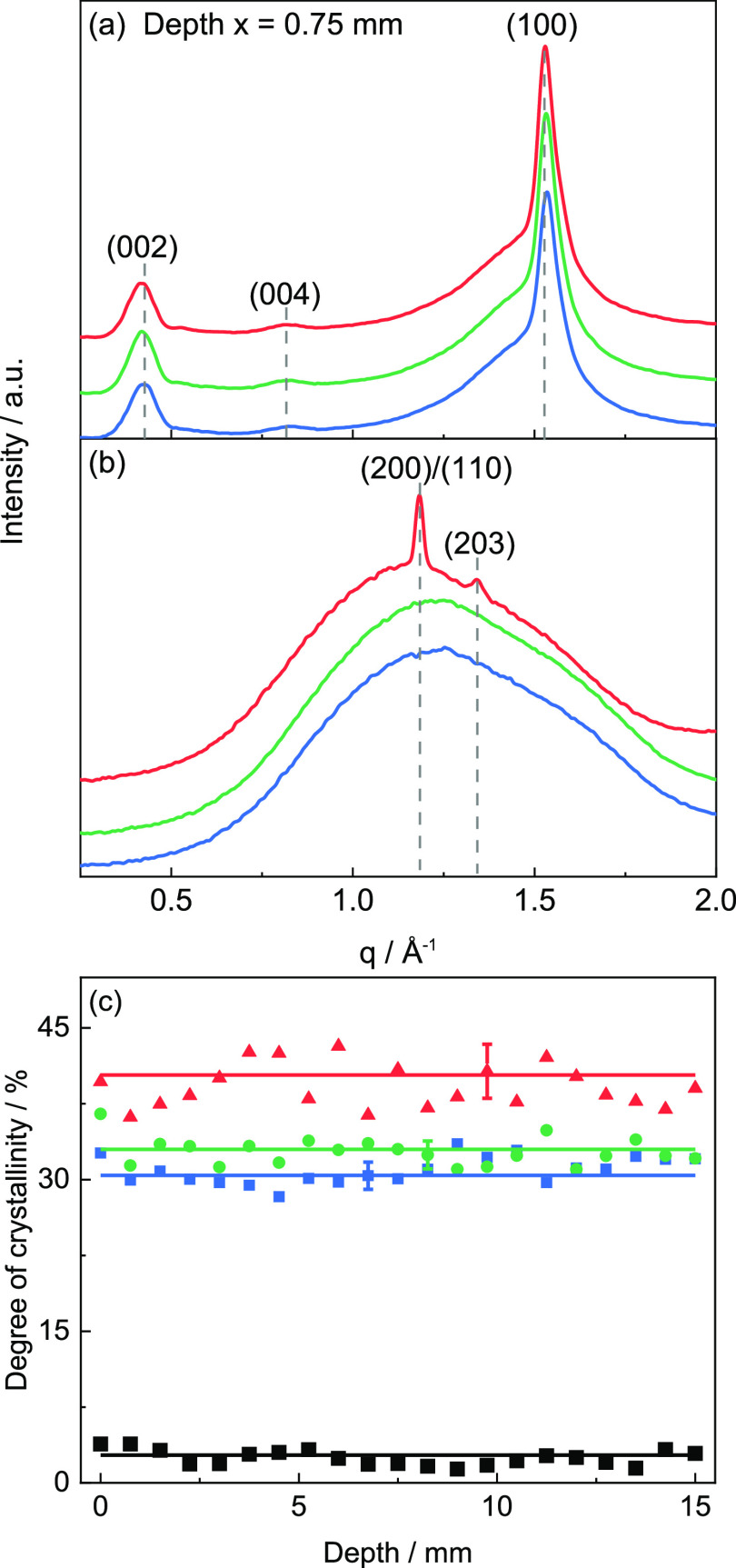
(a) Representative 1D scattering pattern for PA12 single layers for print bed temperatures of *T*_b_ = 30 °C (blue), 80 °C (green), and 120 °C (red). (b) 1D scattering pattern for PLA single layers, printed at print bed temperatures *T*_b_ of 30 °C (blue), 60 °C (green), and 90 °C (red) as obtained at a depth of *x* = 0.75 mm (*y* = 15 mm). (c) Corresponding degree of crystallinity χ_c_ as a function of depth *x* for PA12 single layers as well as for a PLA single layer printed at *T*_b_ = 90 °C (black squares). Average degree of crystallinity  is indicated by dashed lines for each dataset along with the error bar being the standard derivation.

Figure 13a presents a representative azimuthal integrated 1D scattering pattern of 3D-printed PA12 single layers printed at print bed temperatures *T*_b_ of 30, 80, and 120 °C taken at a depth of *x* = 0.75 mm (*y* = 15 mm). The depth-dependent scans are carried out over the entire depth from 0 ≤ *x* ≤ 15 mm in steps of 0.75 mm (*y* = 15 mm). The 1D scattering pattern shows two reflections in the low *q* region range at *q*_002_ = 0.42 Å^–1^ and *q*_004_ = 0.79 Å^–1^ and a major reflection in the WAXD region at *q*_100_ = 1.51 Å^–1^. The corresponding *d*-spacing is *d*_100_ = 4.16 Å. The 1D scattering pattern of PA12 single layers is qualitatively similar to that of the γ phase obtained in temperature-dependent WAXD scans at 30 °C. This leads to the conclusion that the γ phase of PA12 is the preferred phase in the single layers, quasi-independent of the print bed temperature *T*_b_.

The peak fitting analysis is performed with a two peak fit similar to that for the γ phase in temperature-dependent WAXD scans to obtain the area *A*_amo_ and fwhm of the amorphous halo and the Bragg reflection (*A*_*hkl*_ and fwhm_*hkl*_), respectively. Representative values for *q*_100_, *d*_100_, and *L*_100_ are given in Table 4 and are in good agreement with values from temperature-dependent measurements.

**Table 4:** Fit Parameters for 3D-Printed PA12 Single Layers

*T*_b_ [°C]	*q*_100_ [Å^–1^]	*d*_100_ [Å]	*L*_100_ [Å]
30	1.52	4.14	107.2
80	1.54	4.09	109.3
120	1.53	4.11	110.4

Figure 13b shows a representative 1D scattering pattern of the PLA single layers, printed at print bed temperatures *T*_b_ of 30, 60, and 90 °C. The PLA single layers printed at print bed temperatures *T*_b_ of 30 and 60 °C are completely amorphous over the entire depth *x* range between 0 mm < *x* < 15 mm (at *y* = 15 mm). The PLA single layers printed at a print bed temperature *T*_b_ of 90 °C show weak but prominent reflections of the (200)/(110) and (203) planes at *q*_200/110_ = 1.18 Å^–1^ and *q*_203_ = 1.43 Å^–1^. The corresponding *d*-spacings are *d*_200/110_ = 5.32 Å and *d*_203_ = 4.39 Å, i.e., PLA is present in the orthorhombic α phase. Compared to temperature-, height-, and depth-dependent WAXD scans, the intensity of the Bragg reflections is much weaker, and fewer reflections are observed. The average degree of crystallinity  of the PLA single layer at a print bed temperature *T*_b_ of 90 °C is 3 ± 1% (Figure 13c) and is for all depths much lower as compared with the degree of crystallinity χ_c_ of 3D-printed components (about 30%).

In Figure 13c, the degree of crystallinity χ_c_ of the PA12 single layers printed at print bed temperatures *T*_b_ of 30, 80, and 120 °C is given as a function of depth *x*. The average degree of crystallinity  at print bed temperatures *T*_b_ of 30 and 80 °C are 30 and 32% and are in the same range as corresponding values from temperature-dependent measurements for PA12. The average degree of crystallinity  of the PA12 single layer printed at a print bed temperature *T*_b_ of 120 °C is slightly higher (about 40%). However, the observed deviation is within the experimental uncertainties. In contrast to the depth-dependent 2D scattering pattern of the 3D-printed components shown above, all reflections seen for single layers show basically an isotropic intensity distribution.

## Discussion

### Factors Causing Inhomogeneities in the Semi-crystalline State of 3D-Printed Components

#### Formation of Inner Surfaces: Crystallization Times vs Processing times

There are several factors influencing the semicrystalline state of 3D-printed components. Of major importance is the selection of the crystallizable polymer. However, the final semicrystalline state is also heavily affected by the processing conditions, like local cooling rate and shear field. Both factors have to be considered in obtaining components made from semicrystalline polymers with optimum properties and are also most relevant for the occurrence of inhomogeneities in the crystalline state. This applies to semicrystalline polymeric components in general, but in particular to 3D-printed components.

It is expected that material-related aspects and processing conditions are strongly interconnected in the case of 3D-printing. On the one hand, the local cooling rate applied to the molten polymer strand attached to the upper surface of an emerging component is influenced by processing parameters like nozzle temperature *T*_n_, print bed temperature *T*_b_, chamber temperature, processing speed *v* (repetition times), as well as the dimensions of the component. On the other hand, there are material-related influencing factors like crystallization kinetics, heat conductivity, and heat capacity.

**Figure 14:**
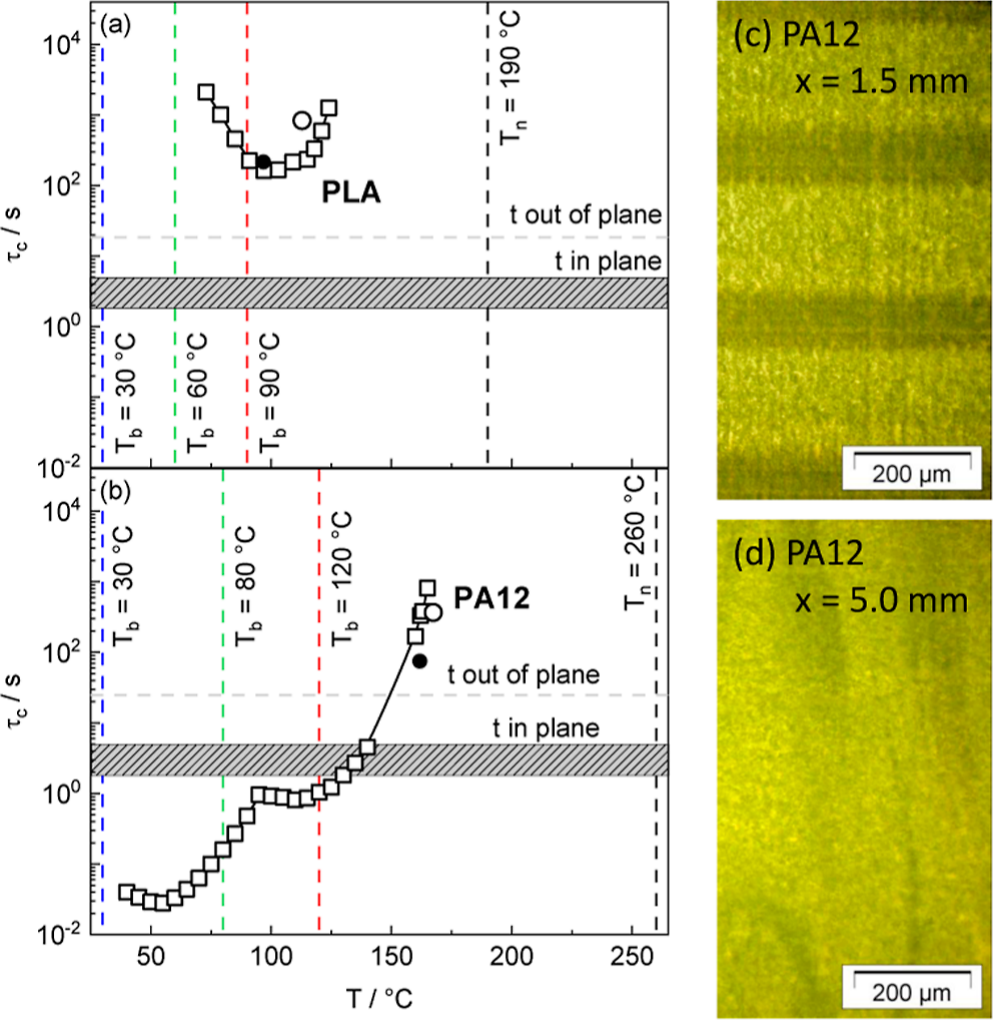
Crystallization times τ_c_ as a function of temperature *T* for (a) PLA and (b) PA12. Nozzle temperature *T*_n_ (black) and bed temperatures *T*_b_ (colored) are shown as vertical dashed lines. Repetition times *t*_r,ip_ and *t*_r,op_ are indicated with horizontal lines. Crystallization times τ_c_ derived from nonisothermal crystallization experiments on PLA^52^ and PA12^53^ in the literature are also given (full circle 5 K/min and open circle 1 K/min). (c,d) POM images of PA12 films taken at a depth of *x* = 1.5 mm (c) and *x* = 5.0 mm (d). PA12 component is printed at a bed temperatures *T*_b_ of 80 °C. Images represent the situation in the middle (*y* = 15 mm) of the films far away from the bed. Prominent interfaces between different layers are visible as thin dark horizontal lines in the outer regions but absent in the core of the component.

From a technical point of view, extremely relevant are times defined by the FFF printing program, like repetition times in-plane *t*_r,ip_ (time it takes to reach a directly neighbored volume element within one layer) and repetition times out-of-plane *t*_r,op_ (time it takes to reach a directly neighbored volume element in the next layer). These processing-related times, *t*_r,ip_ and *t*_r,op_, should determine the interfacial situation to a large extent, i.e., whether or not pronounced interfaces/interphases develop between two neighbored volume elements in a component due to differences in the semicrystalline state. To understand and quantify these effects, it is important to compare the processing-related repetition times, *t*_r,ip_ and *t*_r,op_, with the crystallization times τ_c_ for the chosen polymers. Note that the repetition times were extracted from the G-codes. Figure 14 shows typical repetition times of the used printing program in comparison with crystallization times τ_c_ from isothermal crystallization measurements by calorimetry as a function of temperature *T* for Renkforce PLA and Fiberlogy PA12. Selected results from the literature are given for comparison. Print bed temperatures *T*_b_ and nozzle temperature *T*_n_ are indicated by vertical dashed lines, and repetition times in-plane *t*_r,ip_ and out-of-plane *t*_r,op_ by horizontal lines. In the case of PLA, crystallization is commonly slow. The crystallization times τ_c_ of PLA are significantly longer (at least ten times) than the repetition times *t*_r,ip_ and *t*_r,op_ in the entire temperature range between nozzle *T*_n_ and print bed temperature *T*_b_. One can conclude that crystallization of one volume element will not be completed before a neighbored volume element is printed beside (*t*_r,ip_) or on top (*t*_r,op_). When looking at crystallization times for PA12, it can be seen that PA12 crystallizes much faster compared to PLA. The comparison in Figure 14b shows clearly that crystallization is completed at all investigated print bed temperatures *T*_b_ (30 °C ≤ *T*_b_ ≤ 120 °C) before a new volume element is attached in-plane and out-of-plane. Only at very high temperatures (*T* > 150 °C) are crystallization times τ_c_ longer than relevant processing times *t*_r,ip_ and *t*_r,op_.

To obtain a homogeneous component with minimum differences in the semicrystalline state, crystallization times τ_c_ should be longer than repetition times in-plane and out-of-plane in order to prevent crystallization before neighbored volume elements are printed. This scenario might also be achievable for PA12, but only above a temperature *T* of 150 °C. The conclusion of this comparison is that only for high print bed *T*_b_ and environmental temperature situations can be achieved for PA12, which are similar to the conditions that commonly exist for slowly crystallizing PLA.

The experimental findings of this work support the predictions derived based on Figure 14 and provide further insights. The properties of “isothermally printed components”, i.e., single layers from PLA as considered in Figure 13, are in line with relations between crystallization τ_c_ and processing times *T*_r,ip_ and *t*_r,op_, as highlighted in Figure 14. PLA single layers do not show crystallinity at print bed temperatures *T*_b_ ≤ 90 °C. This can be understood as a consequence of long crystallization times τ_c_ > 150 s for all temperatures ≤90 °C. Since the printing time *t*_r,op_ of a single layer (18 s) is much shorter, one can conclude that crystallization cannot take place before finishing one layer. The very weak degree of crystallinity χ_c_ obtained for the single PLA layer printed at *T*_b_ = 90 °C is somehow expected since the crystallization time τ_c_ is the shortest for this temperature.

The fact that 3D-printed PLA components do show much higher crystallinities away from the print bed (*z* > 5 mm) for all investigated print bed temperatures *T*_b_ (Figure 11) can be interpreted as a consequence of crystallization forced by heat induced by molten material printed on top and by annealing of the component for the time needed to print the entire component layer by layer (3300 s). The temperature in the core region is probably higher, resulting in faster crystallization and in a lower effective cooling rate since the heat introduced by hot material during the printing of subsequent layers is not transported from the core of the component to its outer surfaces or to the print bed due to poor heat conductivity of polymers. Due to the generally slow crystallization kinetics of PLA, a reduction in the degree of crystallinity χ_c_ is for PLA components mainly seen close to the print bed at *T*_b_ ≤ 60 °C where heat can be transferred effectively to the print bed. Away from that region, PLA components exhibit high homogeneity, as crystallization takes place very slowly after several layers have already been printed on top (at least eight layers when assuming a crystallization time τ_c_ of 150 s). In contrast to PLA, PA12 crystallizes much faster under all conditions investigated. The crystallization times τ_c_ are for all print bed temperatures *T*_b_ significantly shorter than the printing time *t*_r,op_ required for one single layer (18 s) as confirmed by the high degree of crystallinity χ_c_ of PA12 single layers printed at print bed temperatures *T*_b_ in the range of 30 °C ≤ *T*_b_ ≤ 120 °C (Figure 11a). The crystallization times τ_c_ below 140 °C are also faster than all repetition times *t*_r,ip_ and *t*_r,op_. This explains why distinct interfaces between different layers are commonly observed for PA12 components printed at bed temperatures *T*_b_ ≤ 120 °C in POM images close to the interface between component/air (Figure 14c). In these cases, the crystallization of one layer is completed before the next layer is printed. This feature disappears only in the core of the component for *T*_b_ ≥ 80 °C where the interfaces between layers vanish (Figure 14d) possibly since the heat introduced by the hot melt leads here together with poor heat conductivity to really high crystallization temperatures in the core region close to 150 °C during 3D-printing. Additional POM images are provided in Supporting Information. The finding that a certain fraction of the α phase is seen in 3D-printed PA12 components supports the idea that crystallization temperatures above 135 °C (being the monoclinic (α/α′) to hexagonal (γ/γ′) transition temperature) occur during the printing of the component.

#### Crystal Orientation Effects: Terminal Relaxation Time vs Crystallization Time

**Figure 15:**
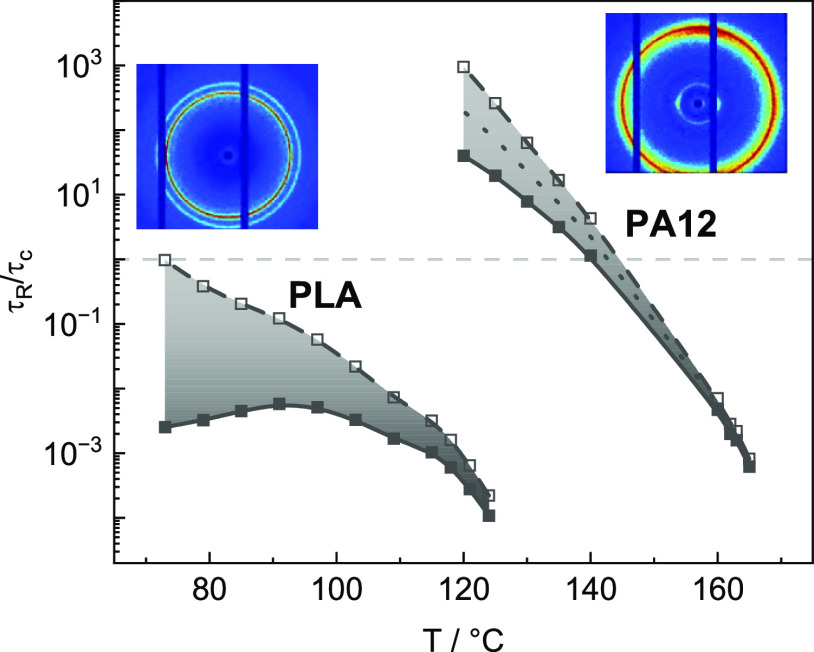
Ratio of relaxation times to crystallization times τ_R_/τ_c_ as a function of temperature *T* of PLA and PA12. The used τ_R_ values are determined based on an Arrhenius extrapolation (full squares, solid line) and alternatively based on a VFTH extrapolation (open symbols, dashed line) like discussed in Figures 7 and 8. Additional dotted line in case of PA12 represents a VFTH extrapolation with *T*_V_ = *T*_g_ – 70 K. Horizontal dashed line is indicating that relaxation takes longer than crystallization if τ_R_/τ_c_ > 1.

Another effect that can be at least partly explained based on the experimental results presented in this work is the existence of differences regarding crystal orientation. For that purpose, it seems to be important to consider the ratio of terminal relaxation time to crystallization time τ_R_/τ_c_ since crystal orientation will not appear without anisotropy in the chain conformation existing before and during crystallization. However, the anisotropy in the chain conformation will disappear before crystallization if the terminal relaxation time is much shorter than the crystallization time for a specific crystallization temperature. Hence, the ratio τ_R_/τ_c_ is plotted as a function of temperature *T* in Figure 15 for PLA and PA12. Also, for this quantity, prominent differences between PLA and PA12 are observed. Isothermal crystallization is for PLA practically always slower than terminal relaxation (onset of flow), while this case occurs for PA12 only at high temperatures above 145 ± 5 °C. At all lower temperatures, crystallization is much faster than the terminal relaxation time. This can explain qualitatively why orientation is commonly absent in 3D-printed PLA components but partly seen for (the α phase of) PA12 components. Main reason that is causing crystal orientations in 3D-printed components should be shear induced in the nozzle region or on the component surface. Since the strands are sheared within the nozzle, polymer chains are stretched and thus potentially oriented during deposition on the bed or component surface.

A ratio τ_R_/τ_c_ < 1 means that the polymer chains have enough time to reach a relaxed state before crystallization. A ratio τ_R_/τ_c_ > 1 means that relaxation cannot take place before crystallization, which results in remaining orientation. In the case of PA12, chain relaxation can only take place at temperatures above 145 ± 5 °C before crystallization occurs. Since α crystals in PA12 components are oriented it can be speculated that the temperature on the upper surface of the components was always below 145 ± 5 °C. Note that we consider here moderate shear rates, where the influence of shear rate  on the flow behavior can still be neglected. In summary, one can conclude that in order to obtain an interface free, more homogeneous, and nonoriented microstructure of PA12 components, it would be reasonable to print at print bed temperatures *T*_b_ above 145 ± 5 °C mimicking the general situation in PLA.

#### Macroscopic Void Formation: Terminal Relaxation Times vs Processing Times

**Figure 16:**
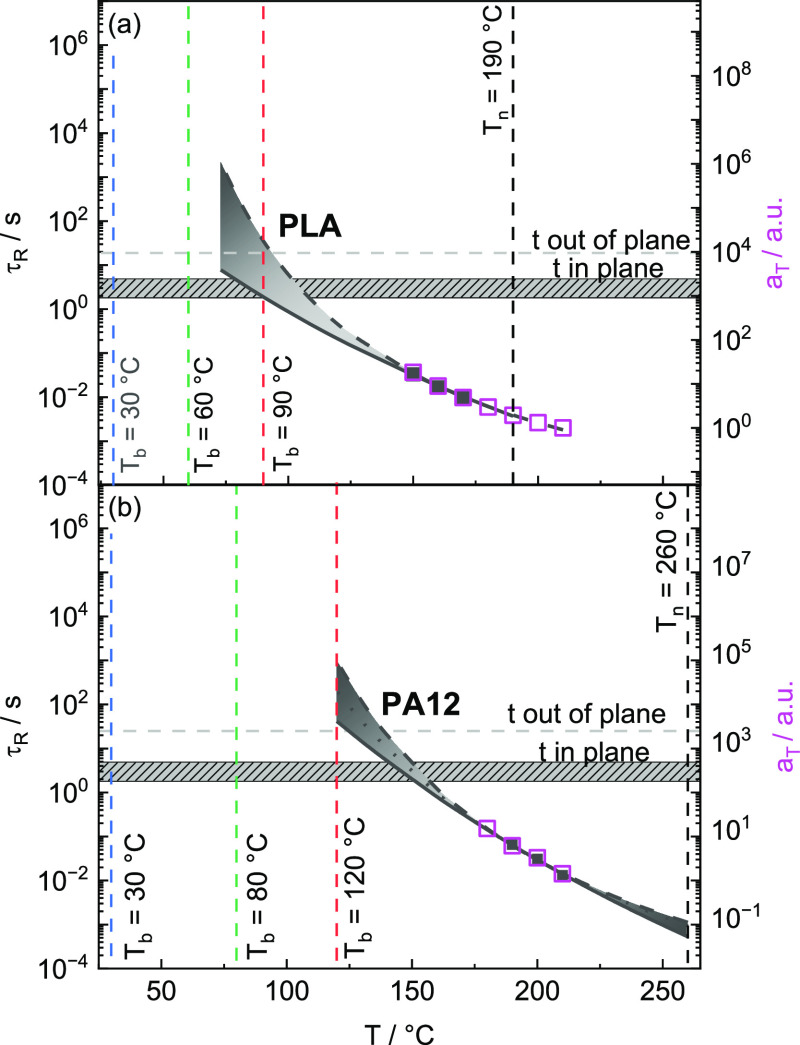
Terminal relaxation times τ_R_ (filled squares) and shift factors *a*_T_ (open squares) as a function of temperature *T* obtained from dynamic mechanical measurements. (a) Extrapolated terminal relaxation times τ_R_ for PLA obtained by an Arrhenius (solid line) and VFTH fit (dashed line). (b) Extrapolated terminal relaxation times τ_R_ for PA12 obtained by an Arrhenius (solid line) and VFTH fit with *T*_V_ = *T*_g_ – 20 K (dashed line) and *T*_V_ = *T*_g_ – 70 K (dotted line). Printing times in-plane and out-of-plane, *t*_r,ip_ and *t*_r,op_, are indicated by horizontal lines. Nozzle *T*_n_ and print bed temperatures *T*_b_ are indicated with vertical dashed lines.

To achieve compact components without macroscopic voids, it is important to know the flow behavior of 3D-printing materials. In the previous section, the influence of terminal relaxation time τ_R_ on the semicrystalline state within a component has been already discussed. However, knowledge of terminal relaxation time is also important to choose the right printing program from the viewpoint of macroscopic void formation and defects. In the literature, sometimes criteria based on viscosity are used, e.g., a zero shear viscosity η_0_ of about 10^2^ to 10^3^ Pa·s.^25−29^ The terminal relaxation time τ_R_ is an alternative since it indicates the transition from a rubbery state to a liquid state. Somehow, this is a more robust quantity since well-defined viscosities are often hard to get for commercial polymers at application relevant temperatures. The rubber to liquid transition is not sharp but a continuous transition since (in particular for commercial grades with broad molecular weight distribution) relaxation features play an important role in the flow transition range. Figure 16 presents terminal relaxation times τ_R_ for PLA and PA12 as a function of temperature *T* in comparison with in-plane and out-of-plane repetition times *t*_r,ip_ and *t*_r,op_. To extend the temperature interval, terminal relaxation times obtained from dynamic mechanical measurements are extrapolated based on the Arrhenius law and a VFTH estimation.

The temperature dependence of PA12 is seemingly more pronounced compared to that of PLA, and the terminal relaxation time τ_R_ at the nozzle temperature *T*_n_ is significantly shorter (indicating lower viscosity η). In principle, the molten filament strand should flow only for a certain time period after deposition on the surface of the component. Which shape is observed and to what extent void formation can be excluded depend to a large extent on the cooling behavior. Flow stops if a temperature is reached where the terminal relaxation times τ_R_ are too long or if crystallization occurs. In a way, one can argue that temperatures where τ_R_ > *t*_r,ip_ should be irrelevant for 3D-printing conditions since an appropriately shaped strand on the surface should be formed before the nozzle is printing the next strand beside it. Strand shaping on the surface should be completed above that temperature where τ_R_ = *t*_r,ip_ (cf., Figure 16) if shear rate-dependent effects are neglected. Crystallization during cooling seems to appear under relevant application conditions, usually at a lower temperature for both investigated polymers. Importantly, all the details depend on the local cooling rate.

Insufficient flow can lead to processing errors, such as macroscopic voids. Henceforth, to avoid voids and get a smooth surface, it is important to optimize the flow behavior of polymeric filaments, knowing the relevant conditions of the 3D-printing process.

## Conclusions

Summarizing the above discussion about influencing factors, one can conclude that producing 3D-printed components with a small number of intrinsic interfaces and homogeneous semicrystalline structure requires a detailed knowledge of terminal relaxation and crystallization times (τ_R_, τ_c_) of the used material and processing-related times like repetition times in-plane and out-of-plane (*t*_r,ip_ and *t*_r,op_). For 3D-printed components with optimal properties, different ratios of these times should be considered or incorporated in numerical simulations in order to find suitable strategies to improve component quality. In particular, one should consider the following guidelines:

(1) to get a spatially uniform semicrystalline state without pronounced interfaces within a component, one should tune the repetition times (within a layer or layer-to-layer) during printing in such a way that repetition times (*t*_r,ip_ and *t*_r,op_) are shorter than crystallization times τ_c_(*T*, ) under printing conditions; accordingly the printing speed *v* should be adapted ideally to the crystallization kinetics of the chosen material (or vice versa, e.g., by adding/removing nucleating agents to/from the used polymer).
(2) to avoid anisotropy of the semicrystalline structures within a 3D-printed component, one should choose printing conditions where the ratio τ_R_/τ_c_ is smaller than one; this means that shear stresses can relax to a large extent before crystallization begins; the situation can be influenced, e.g., by temperature conditions and molecular weight of the chosen polymer or additivation.
(3) to prevent void formation and related defects of 3D-printed components, one should choose printing conditions that fit to the terminal relaxation time τ_R_ and overall flow behavior of the polymer filament used; the nozzle temperature *T*_n_ is important here, the shape of the strands formed on the component surface depends on it, but also the subsequent cooling process and the shear field in the nozzle are important; process-based strategies to improve the situation are changes in nozzle *T*_n_, print bed *T*_b_, and chamber temperature or a material-based approach focusing, for example, on the optimization of the molecular weight distribution.

These guidelines show that for a problem-specific optimization, a detailed knowledge of relevant material properties, cooling conditions, and shear introduced in the nozzle is important. While material-related parameters can be measured by suitable methods, the process-specific information can in detail be provided only by numerical simulations. Therefore, suitable input parameters should be determined and used in order to be able to choose optimized materials and processing conditions for each individual polymeric component to be produced by FFF.

## Supporting Information Available

Temperature-dependent WAXD measurements for PA12 and PLA filaments, peak deconvolution procedure of the 1D WAXD pattern, shift factors and terminal relaxation times from the DMA measurements, and structural characterization of the 3D-printed components by POM (PDF)
