# Supplementary material for: Influence of Crystallization Kinetics and Flow Behavior on Structural Inhomogeneities in 3D-Printed Parts Made from Semi-Crystalline Polymers
Source: Macromolecules. 2024 Mar 19;57(7):3066–80. doi: 10.1021/acs.macromol.3c01940 (PMC11008537; doi:10.1021/acs.macromol.3c01940)
Supplement: Supplementary file 1 — ma3c01940_si_001.pdf [file ma3c01940_si_001.pdf]

Influence of crystallization kinetics and flow behavior on structural inhomogeneities in 3D printed parts made from semi-crystalline polymers
Supporting Information

Rene Sattler,[†,‡] Rui Zhang,[¶] Gaurav Gupta,[†,‡] Mengxue Du,[¶] Paul-Maximilian Runge,[§] Holm Altenbach,[§] René Androsch,[¶] and Mario Beiner[*,†,‡]

†*Fraunhofer Institute for Mikrostructure of Materials and Systems IMWS, Walter-Hülse-Str. 1, DE-06120 Halle (Saale)*

‡*Faculty of Natural Sciences II, Martin-Luther-University Halle-Wittenberg, Heinrich-Damerow-Str. 4, D-06120 Halle (Saale)*

¶*Interdisciplinary Center for Transfer-Oriented Research in Natural Sciences, Martin-Luther-University Halle-Wittenberg, Universitätsplatz 10, D-06108 Halle (Saale)*

§*Institute of Mechanics, Otto-von-Guericke-University Magdeburg, Universitätsplatz 2, D-39106 Magdeburg*

E-mail: mario.beiner@imws.fraunhofer.de

Temperature-dependent structure formation in the bulk state for PA12 and PLA filaments

PA12. Figure SI1 presents the azimuthal integrated 1D scattering pattern of PA12 obtained during step wise cooling and 2nd heating run. Crystallization starts from 160 °C. At 150 °C, two Bragg reflections indexed as q_{200} (q_{200} = 1.44 Å^{-1}) and q_{010} (q_{010}= 1.48 Å^{-1}), corresponding to the α' phase[1–7] (monoclinic unit cell), are observed in the WAXD region during cooling. The corresponding d-spacing are d_{200} = 4.36 Å and d_{010} = 4.25 Å, respectively. Upon further cooling, q_{200} is shifting to higher q-values, resulting in a merger of q_{200} and q_{010} at 130 °C to a single sharp reflection at q_{100} = 1.51 Å^{-1} (d_{100} = 4.15 Å). The single strong reflection at ≈ 1.5 Å^{-1} is associated to the (pseudo)hexagonal γ phase,[4–7] observed between 130 °C ≤ T ≤ 30 °C. In the low q region two reflections are observed at q_{002} = 0.42 Å^{-1} and q_{004} = 0.80 Å^{-1}. The (002) along with it's higher order (004) planes correspond to the periodicity of the amide bonds along the main chain. The scattering pattern of the 2nd heating run of PA12 are qualitatively similar. In order to obtain the temperature-dependent

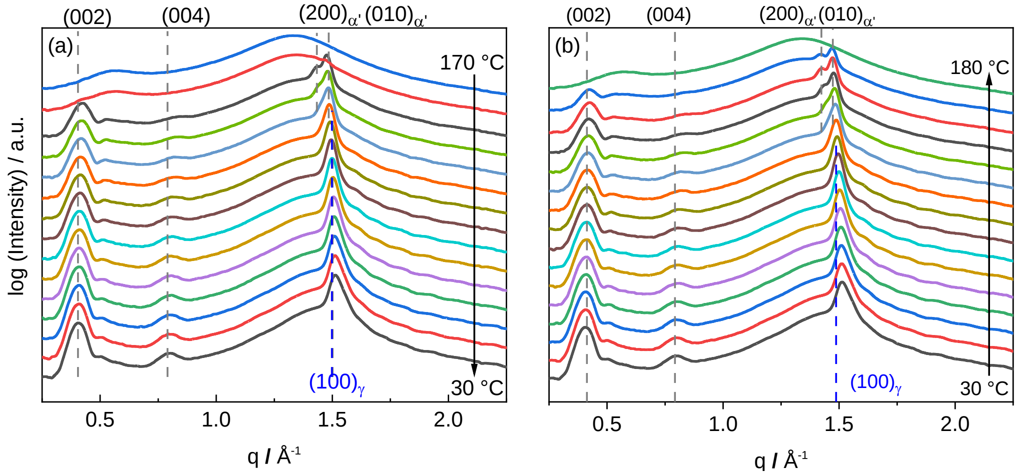

Figure SI 1: Temperature-dependent azimuthal integrated 1D scattering pattern of PA12 during (a) step wise cooling from the relaxed melt and (b) 2nd heating run. The temperature interval was between 30 °C and 220 °C, the rate used was ±10 K/min between each measurement step. Major reflections are indicated.

locations $q_{\rm hkl}$ as well as area $A_{\rm hkl}$ of the Bragg reflections and amorphous halo $A_{\rm amo}$ a detailed

peak analysis is applied. Representative examples of the deconvoluted 1D scattering pattern in the WAXD region are shown in Figure SI2 for different polymorphic states. In Figure SI3

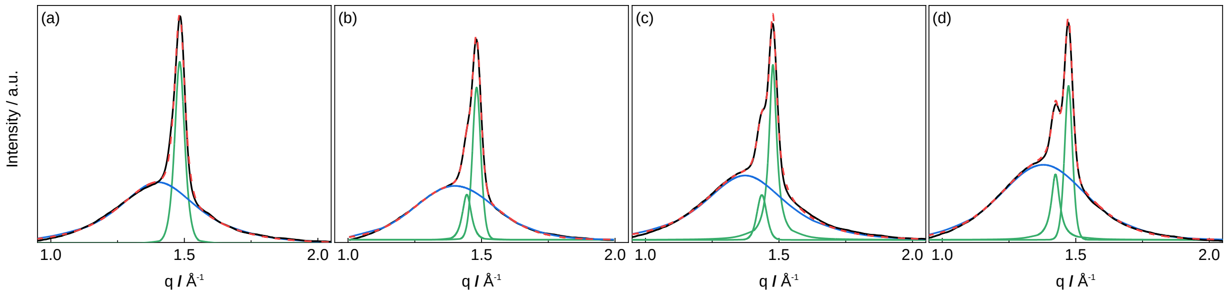

Figure SI 2: Representative examples of the peak deconvolution. PA12 in the (a) (pseudo)hexagonal γ phase at 30 °C, (b) mesophase consisting of γ and α' phase at 140 °C and (c) 160 °C and (d) the monoclinic high temperature α' phase at 180 °C. The baseline corrected 1D scattering pattern are shown in black, Bragg reflections in green, amorphous halo in blue and cumulative fit in red dashed.

the d-spacing and degree of crystallinity χ_c are plotted as function of temperature T during cooling and 2nd heating run. The $\alpha' \leftrightarrow \gamma$ phase transition is a reversible solid-solid transition[5] as evidenced by the evolution of the d-spacing in the WAXD region during cooling and subsequent 2nd heating run.

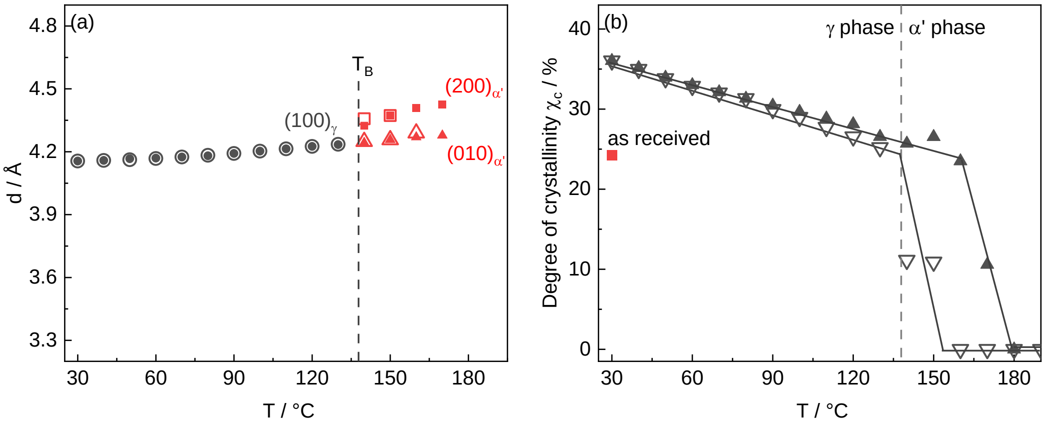

Figure SI 3: Temperature-dependence of the (a) d-spacing and (b) degree of crystallinity χ_c during cooling (open symbols) and subsequent 2nd heating run (full symbols). The degree of crystallinity χ_c of as received PA12 at room temperature is shown as red square. The Brill transition temperature T_B is indexed with a dashed line.

PLA. The azimuthal integrated 1D scattering pattern of PLA obtained during step wise cooling and subsequent 2nd heating run are shown in Figure SI4. Qualitatively, the scattering pattern measured during the 2nd heating run are similarly exhibiting a single polymorphic state, i.e. the orthorhombic α phase. Crystallization during cooling run starts from 120 °C where two major Bragg reflections occur in WAXD region at $q_{200/110}$ = 1.175 Å^{-1} and q_{203} = 1.335 Å^{-1} ($d_{200/110}$ = 5.32 Å and d_{203} = 4.65 Å). Below 110 °C several reflections arise which are all corresponding to the orthorhombic α phase (a = 10.62 Å, b = 6.13 Å and c = 28.95 Å) of PLA.[1–3] In order to obtain the locations q_{hkl} as well as area A_{hkl} of the Bragg

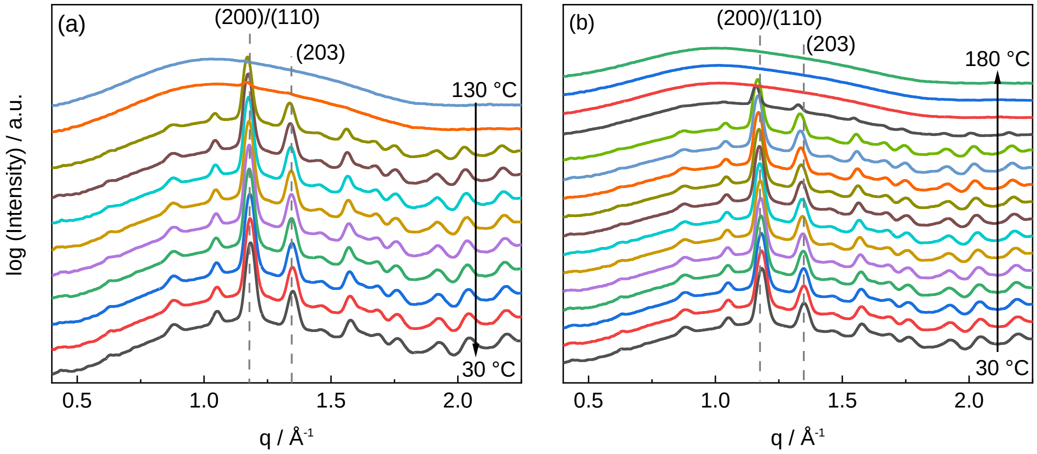

Figure SI 4: Temperature-dependent azimuthal integrated 1D scattering pattern of PLA during (a) step wise cooling and (b) subsequent 2nd heating run. The temperature interval was between 30 °C to 180 °C, the rate between the measurement steps was ±10 K/min. The two major reflections are indexed. Further weak reflections are indexed in Figure SI5.

reflections and amorphous halo A_{amo} a detailed peak analysis is applied as shown in Figure SI5. Figure SI6 presents the temperature-dependence of the major d-spacing $d_{200/110}$ and d_{203} and the degree of crystallinity χ_{c} as function of temperature T during cooling and 2nd heating run.

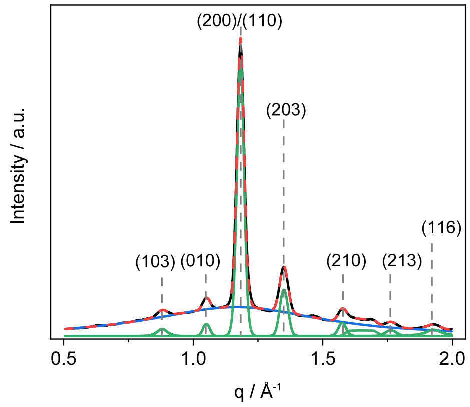

Figure SI 5: Representative example of peak deconvolution for the 1D scattering pattern of PLA in the orthorhombic α phase after slow cooling from the molten state. The baseline corrected 1D scattering data is shown in black, Bragg reflections in green, amorphous halo in blue and cumulative fit in red dashed. All reflections are indexed

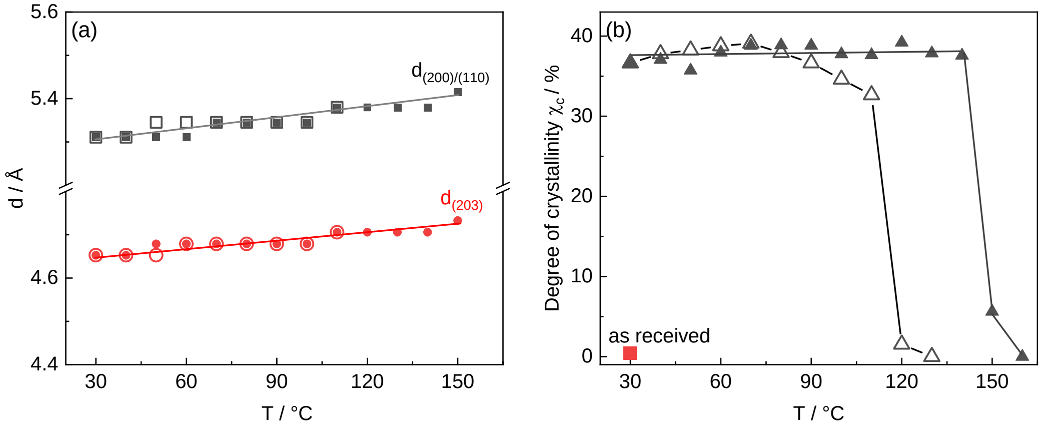

Figure SI 6: Temperature dependent evolution of the (a) two major d-spacing and (b) degree of crystallinity χ_c during cooling (open symbols) and 2nd heating run (full symbols). The degree of crystallinity χ_c of as received PLA at room temperature is shown as red square.

Results from Dynamic Mechanical Analysis

Terminal relaxation times τ_{R} as well as horizontal shift factors a_{T} from a master curve construction are compiled in Table SI1 for the PA12 and PLA filaments.

Table SI 1: Terminal relaxation times τ_{R} and shift factors a_{T} for PA12 and PLA

PA12			
T [°C]	$1000/T$ [K^{-1}]	τ_{R} [s]	a_{T} [a.u.]
210	2.07	0.01	1.00
200	2.11	0.03	2.30
190	2.16	0.06	4.29
180	2.21	0.23	10.59
PLA			
210	2.07	-	1.00
200	2.11	-	1.31
190	2.16	-	1.93
180	2.21	-	2.98
170	2.26	0.01	4.88
160	2.31	0.02	8.89
150	2.36	0.03	18.31

Peak analysis of scattering pattern of 3D printed components

Figure SI7 presents the deconvoluted scattering pattern of FFF printed PA12 and PLA components. While the scattering pattern of PLA are qualitatively similar to that obtained during step wise cooling, clear differences can be observed in case of PA12. Besides the strong reflection at ≈ 1.5 Å^{-1}, corresponding to the (pseudo)hexagonal γ phase, two shoulders at lower and higher q-values occur. A peak deconvolution reveals two additional Bragg reflections, most likely caused by a mixture between γ and α phases.

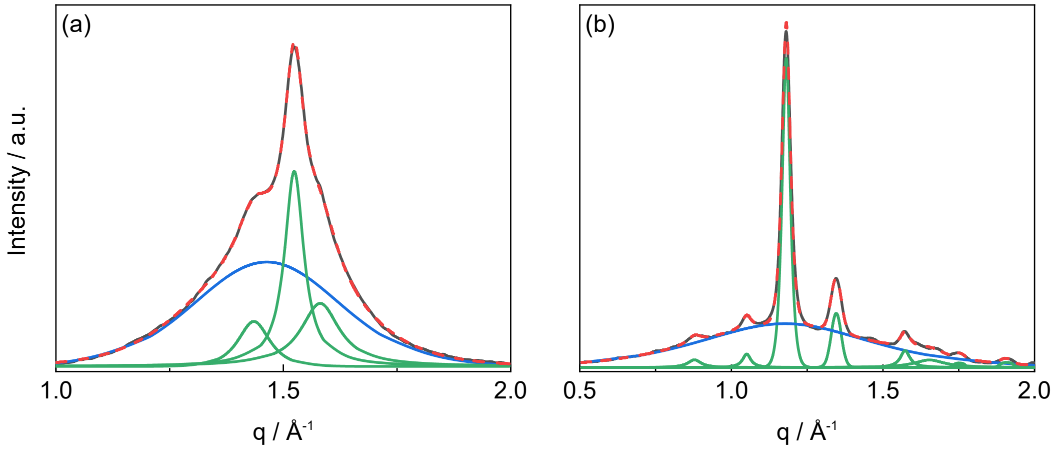

Figure SI 7: Representative examples of deconvoluted scattering pattern of 3D printed components made of (a) PA12 and (b) PLA. The baseline corrected 1D scattering data is shown in black, Bragg reflections in green, amorphous halo in blue and cumulative fit in red dashed.

Structural characterization of 3D-printed components by Polarized Optical Light Microscopy

PA12. Polarized Light Optical Microscopy (POM) images are captured using a Olympus BX51 polarized light microscope with AnalySIS software. The thin films are mounted with double-sided tape on slides to ensure a as flat as possible surface. To study how the structure changes by approaching the core, thin films are examined at similar heights and depths as in the WAXD measurements. Height dependent POM measurements with 5k magnification are performed on thin PA12 films taken from components printed at bed temperatures T_b of 30 °C, 80 °C and 120 °C. Thin films microtomed in a depth x of 2.5 mm from the lower filled part of components are investigated. Figure SI8 presents POM images captured at heights z of 0.6 mm and 10.2 mm. In all cases a layered morphology due to 3D-printing is observed. The layers correspond to the height of an individually printed layer z_l (about 0.3 mm) indicating that the printing process causes significant structural heterogeneities. A clear influence of the bed temperature T_b as well as distance z from the bed on the morphology can be observed. With increasing bed temperature T_b and distance from the bed z, the inter-facial regions are less pronounced and the layers become more homogeneous. In an addition the depth

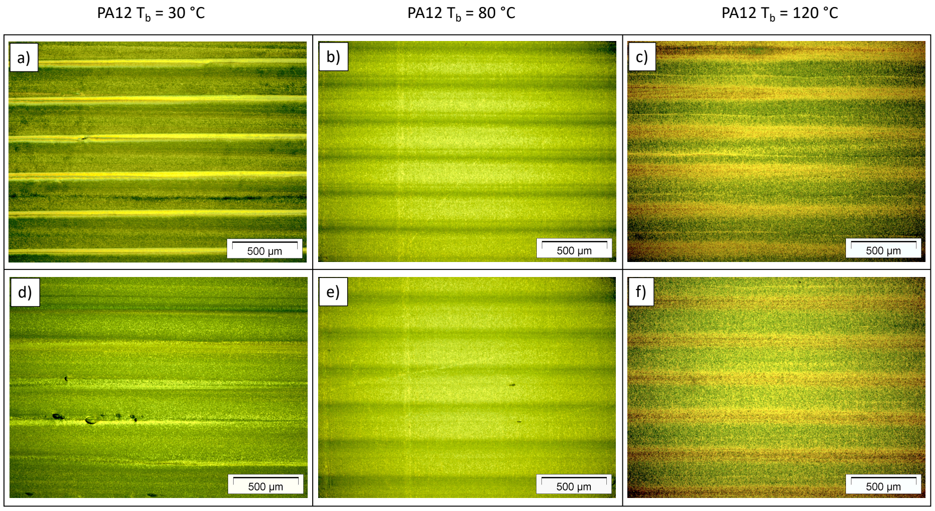

Figure SI 8: POM images (magnification 5k) of PA12 thin films taken at a depth x of 2.5 mm from components printed with bed temperatures T_{b} of 30 °C, 80 °C and 120 °C. The images are taken at a height z of 0.6 mm (top) and 10.2 mm (bottom) in the middle of the film (y = 15 mm). The interfaces between individually printed layers are visible as dark horizontal lines.

dependence of the morphology in POM images is studied at depths x of 1.5 mm and 5.0 mm. Figure SI9 shows images with 10 k magnification of thin films microtomed at different depth from PA12 components printed at bed temperatures T_{b} of 30 °C, 80 °C and 120 °C. For a depth x of 1.5 mm one can clearly see an improvement of homogeneity with increasing bed temperature T_{b}, i.e. the interfaces between layers become less pronounced. In addition the homogeneity is improving by approaching the core of the component. At a bed temperature T_{b} of 30 °C, the interfaces between individual layers are still visible at a depth x of 5.0 mm. A direct comparison of the images for the components printed at bed temperatures T_{b} of 80 °C or 120 °C demonstrates a higher degree of homogeneity, where the interfaces between printed layers are no longer visible. Components printed at a bed temperature T_{b} of 120 °C show distinctive voids at a depth x of 5.0mm (Figure SI9 (f)).

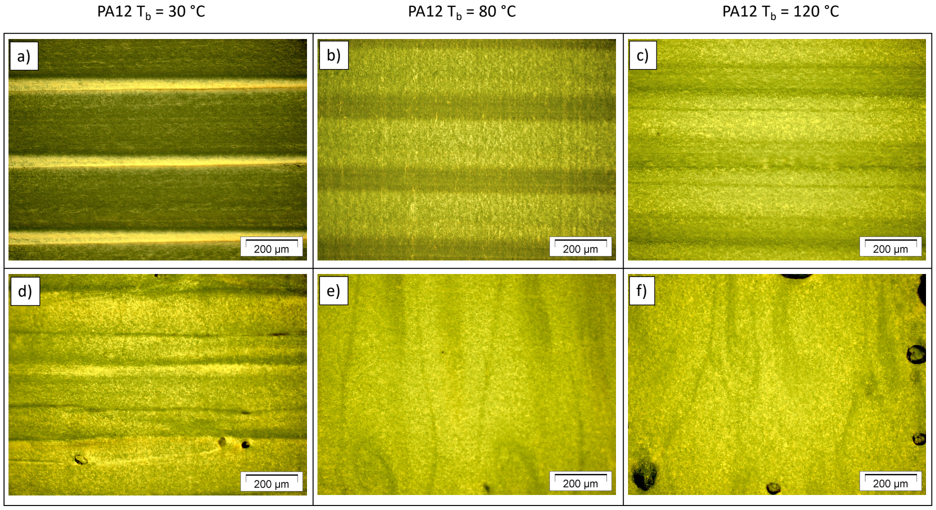

Figure SI 9: POM images (magnification 10k) of PA12 films taken from components printed at bed temperatures T_b of 30 °C, 80 °C and 120 °C at a depth x of 1.5 mm (top) and 5.0 mm (bottom). The images represent the situation in middle of the films ($y = 15$ mm). Interfaces between layers are in some cases visible as thin dark horizontal lines.

PLA. POM measurements with 5k magnification are performed at different heights z on thin films microtomed from PLA components printed at bed temperatures T_b of 30 °C, 60 °C and 90 °C. The films are taken in a depth x of 2.5 mm from the filled part of the components. Figure SI10 presents POM images obtained at heights z of 0.6 mm and 10.2 mm. Similar to the POM images of PA12 thin films, PLA thin films show interfaces between the printed layers indicated by thin dark horizontal lines which become less pronounced with increasing bed temperature T_b and height z. However, the interfaces are seemingly less extended compared as to PA12 and the components appear in general slightly more homogeneous. The additional features seen in the POM images either as vertical or horizontal grooves are cutting artifacts resulting from microtomy. The variation of the direction of the surface artifacts in Figure SI10(c,f) is due to changes in the clamping direction. The features are not related to internal structures formed during 3D-printing. Depth dependent POM images captured at depths x of 1.5 mm and 5.0 mm support the findings reported for the thin films taken from a depth x of 2.5 mm. In figure SI11 POM images measured on thin films taken in a depth x of 1.5 mm and 5.0 mm from components printed at different bed temperatures

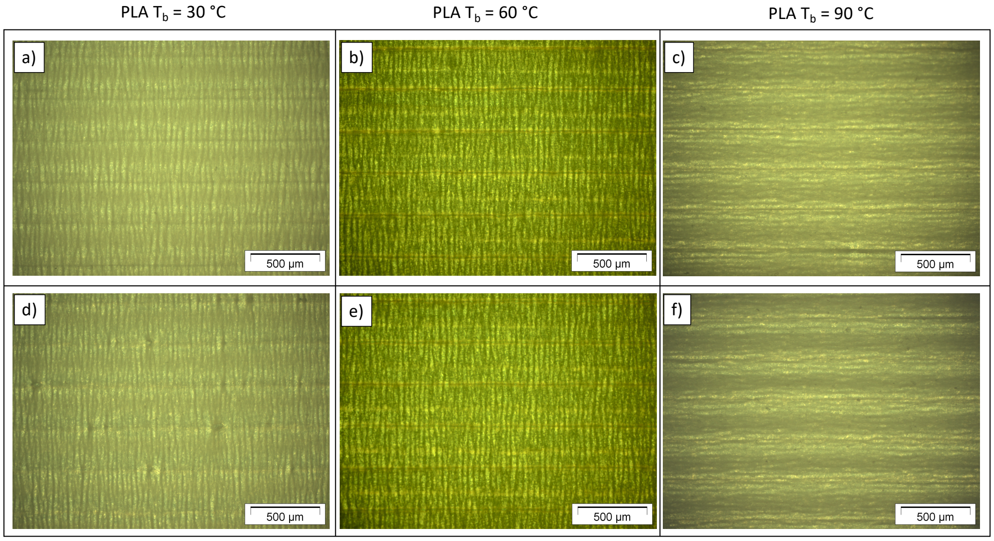

Figure SI 10: POM images (magnification 5k) of PLA thin films with bed temperature Figure SI8 of 30 °C, 80 °C and 120 °C at heights z of 0.6 mm (top) and 10.2 mm (bottom). The images represent the situation in middle of the films (y = 15 mm).

T_b are compared. PLA components show well pronounced interfaces between layers for at all bed temperatures T_b at depth x of 1.5 mm. This is may be caused by a severe temperature gradient between core (high temperatures near melting temperature) and skin layer (lower ambient temperature of chamber). Since PLA has very slow crystallization kinetics, lower temperatures near the skin layer may lead to premature solidification without sufficient fusion of layers. At a depth x of 5.0 mm the PLA component printed at a print bed temperature T_b of 30 °C still shows remaining but less pronounced interfaces between individual layers. PLA components printed at bed temperatures T_b of 80 °C or 120 °C respectively (Figure SI11(e,f)) show a nearly homogeneous structure without clearly detectable interfaces between the layers. Note that in case of PLA with a bed temperature of $T_b = 90$ °C sample was rotated by 90°.

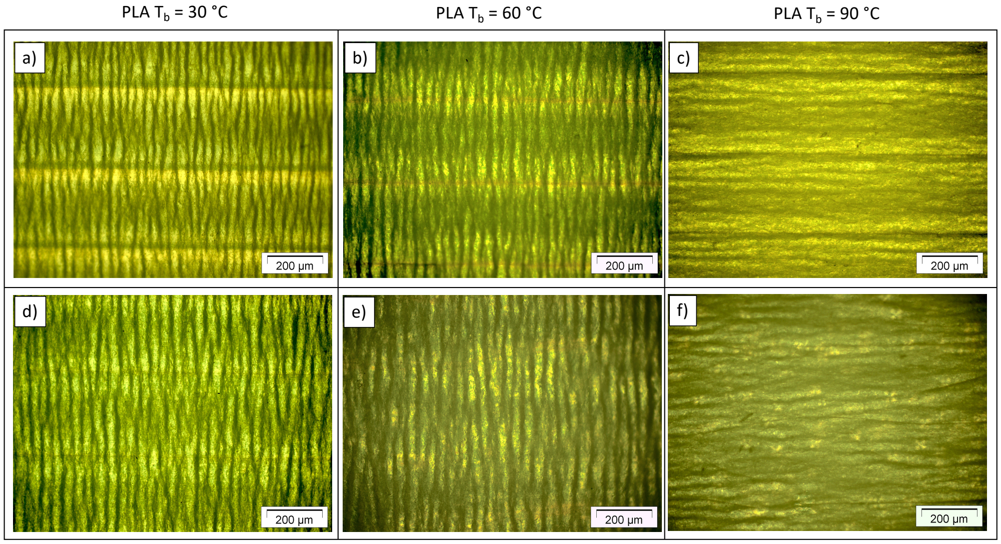

Figure SI 11: POM images (magnification 10k) of PLA films taken at depths x of 1.5 mm (top) and 5.0 mm (bottom) from components printed with bed temperatures T_b of 30 °C, 80 °C and 120 °C. The images represent the situation in middle of the films (y = 15 mm).

References

(1) Kawai, T.; Rahman, N.; Matsuba, G.; Nishida, K.; Kanaya, T.; Nakano, M.; Okamoto, H.; Kawada, J.; Usuki, A.; Honma, N.; Nakajima, K.; Matsuda, M. Crystallization and Melting Behavior of Poly (L-lactic Acid). *Macromolecules* **2007**, *40*, 9463–9469.

(2) Wasanasuk, K.; Tashiro, K. Crystal structure and disorder in Poly(L-lactic acid) δ form (α' form) and the phase transition mechanism to the ordered α form. *Polymer* **2011**, *52*, 6097–6109.

(3) Lohmeijer, P. J. A.; Goossens, J. G. P.; Peters, G. W. M. Quiescent crystallization of poly(lactic acid) studied by optical microscopy and light-scattering techniques. *Journal of Applied Polymer Science* **2017**, *134*, 44566.

(4) Ramesh, C. Crystalline Transitions in Nylon 12. *Macromolecules* **1999**, *32*, 5704–5706.

(5) Li, L.; Koch, M. H. J.; de Jeu, W. H. Crystalline Structure and Morphology in Nylon 12: A Small- and Wide-Angle X-ray Scattering Study. *Macromolecules* **2003**, *36*, 1626–1632.

(6) Dencheva, N.; Nunes, T. G.; Oliveira, M. J.; Denchev, Z. Crystalline structure of polyamide 12 as revealed by solid-state 13C NMR and synchrotron WAXS and SAXS. *Journal of Polymer Science Part B: Polymer Physics* **2005**, *43*, 3720–3733.

(7) Fischer, C.; Seefried, A.; Drummer, D. Crystallization and Component Properties of Polyamide 12 at Processing-Relevant Cooling Conditions. *Polymer Engineering & Science* **2017**, *57*, 450–457.
